# Putative critical quality attribute matrix identifies mesenchymal stromal cells with potent immunomodulatory and angiogenic “fitness” ranges in response to culture process parameters

**DOI:** 10.3389/fimmu.2022.972095

**Published:** 2022-11-30

**Authors:** Kevin P. Robb, Julie Audet, Rajiv Gandhi, Sowmya Viswanathan

**Affiliations:** ^1^ Osteoarthritis Research Program, Division of Orthopedic Surgery, Schroeder Arthritis Institute, University Health Network, Toronto, ON, Canada; ^2^ Krembil Research Institute, University Health Network, Toronto, ON, Canada; ^3^ Institute of Biomedical Engineering, University of Toronto, Toronto, ON, Canada; ^4^ Department of Surgery, Division of Orthopedic Surgery, University of Toronto, Toronto, ON, Canada; ^5^ Department of Medicine, Division of Hematology, University of Toronto, Toronto, ON, Canada

**Keywords:** mesenchymal stromal cell, immunomodulation, angiogenesis, critical quality attribute, donor heterogeneity, 3D suspension cultures, hypoxic conditioning, potency

## Abstract

Adipose-derived mesenchymal stromal cells (MSC(AT)) display immunomodulatory and angiogenic properties, but an improved understanding of quantitative critical quality attributes (CQAs) that inform basal MSC(AT) fitness ranges for immunomodulatory and/or angiogenic applications is urgently needed for effective clinical translation. We constructed an *in vitro* matrix of multivariate readouts to identify putative CQAs that were sensitive enough to discriminate between specific critical processing parameters (CPPs) chosen for their ability to enhance MSC immunomodulatory and angiogenic potencies, with consideration for donor heterogeneity. We compared 3D aggregate culture conditions (3D normoxic, 3D-N) and 2D hypoxic (2D-H) culture as non-genetic CPP conditions that augment immunomodulatory and angiogenic fitness of MSC(AT). We measured multivariate panels of curated genes, soluble factors, and morphometric features for MSC(AT) cultured under varying CPP and licensing conditions, and we benchmarked these against two functional and therapeutically relevant anchor assays – *in vitro* monocyte/macrophage (MΦ) polarization and *in vitro* angiogenesis. Our results showed that varying CPP conditions was the primary driver of MSC(AT) immunomodulatory fitness; 3D-N conditions induced greater MSC(AT)-mediated MΦ polarization toward inflammation-resolving subtypes. In contrast, donor heterogeneity was the primary driver of MSC(AT) angiogenic fitness. Our analysis further revealed panels of putative CQAs with minimum and maximum values that consisted of twenty MSC(AT) characteristics that informed immunomodulatory fitness ranges, and ten MSC(AT) characteristics that informed angiogenic fitness ranges. Interestingly, many of the putative CQAs consisted of angiogenic genes or soluble factors that were inversely correlated with immunomodulatory functions (*THBS1, CCN2, EDN1, PDGFA*, *VEGFA*, *EDIL3*, *ANGPT1*, and *ANG* genes), and positively correlated to angiogenic functions (VEGF protein), respectively. We applied desirability analysis to empirically rank the putative CQAs for MSC(AT) under varying CPP conditions and donors to numerically identify the desirable CPP conditions or donors with maximal MSC(AT) immunomodulatory and/or angiogenic fitness. Taken together, our approach enabled combinatorial analysis of the matrix of multivariate readouts to provide putative quantitative CQAs that were sensitive to variations in select CPPs that enhance MSC immunomodulatory/angiogenic potency, and donor heterogeneity. These putative CQAs may be used to prospectively screen potent MSC(AT) donors or cell culture conditions to optimize for desired basal MSC(AT) immunomodulatory or angiogenic fitness.

## Introduction

The immunomodulatory and pro-angiogenic functions of mesenchymal stromal cells (MSCs) make them attractive cell therapy candidates for numerous clinical indications (1). However, despite hundreds of clinical trials, mixed reports on clinical efficacy and insufficient characterization of MSC potency have continued to hamper the field, resulting in very few MSC products with regulatory and market endorsement (2). As was recently outlined by Krampera and Le Blanc (3), part of the observed heterogeneity in clinical efficacy of MSCs is likely due to the complex interactions between MSCs and the host tissue microenvironment, which is specific to a given disease as well as stage of the disease. This has further pointed to the need for defining critical quality attributes (CQAs) with established limits or ranges that enable quality checks of basal thresholds for high therapeutic potency MSCs with associated fitness levels (4). These fitness levels may be further modulated by the host disease microenvironment to ultimately determine net MSC therapeutic efficacy (3, 5), but quantifying basal MSC fitness through CQAs is a necessary starting point. Importantly, MSC CQAs must be linked to specific culture conditions, which are a critical source of variability in processing and expanding MSCs (6, 7).

Previous work has investigated MSC characteristics that carry functional significance and can serve as candidate CQAs for defining basal MSC fitness levels that correlate with clinical efficacy. Our group has demonstrated that prevalence of a panel of seven immunomodulatory markers expressed by bone marrow-derived MSCs (MSC(M)) *in vitro* (i.e., basal CQAs) correlated with improved patient-reported outcomes, suggestive of an anti-inflammatory mechanism of action in a twelve-patient knee osteoarthritis trial (8). Work by Galleu *et al.* has suggested that MSC(M) that are more susceptive to host cytotoxic activity afforded better clinical responses in graft-versus-host disease (GVHD) patients (9). Several groups have evaluated multi-dimensional characteristics of MSCs that could be considered putative CQAs, as these correlate to *in vitro* immunosuppression of T cell functions; However, the associations between this immunomodulatory ability and clinical efficacy, at least in GVHD patients, has not panned out (10). Nonetheless, work by Chinnadurai *et al.* demonstrated that interactions with peripheral blood-derived mononuclear cells (PBMCs) modulate the mRNA and secreted factor profiles (11, 12), as well as the signal transducer and activator of transcription (STAT) phosphorylation status (13) of human MSCs derived from various tissue sources, and that these signatures correlate with T cell immunosuppression. Maughon *et al.* have shown that metabolomic and cytokine profiles of human MSC(M) and induced pluripotent stem cell-derived MSCs also correlate to T cell suppression (14). Furthermore, multidimensional profiles of human MSC(M) morphological features have been linked to T cell suppression for MSC(M) stimulated with TNFα and/or IFNγ (15, 16). Notably, work by Boregowda *et al.* postulated a potential interplay between immunomodulatory and pro-angiogenic functions of human MSC(M) mediated by expression levels of the transcription factor TWIST1, and suggested that culture conditions impact the interplay between these two properties of MSCs (17).

While progress has been made in identifying candidate CQAs for MSCs (18, 19), it is important for these candidate CQAs to be measurable, quantitative and sensitive to donor heterogeneity and variations in critical processing parameters (CPPs, *i.e*., culture parameters that influence CQAs), two major, controllable variables that impact MSC functional activity *in vitro*. Subsequent *in vivo* MSC functionality is less tractable and likely modulated by host immune cell and microenvironment interactions. Donor heterogeneity can be attributed to several factors, including donor health status, BMI, sex, and age which are known to influence MSC functional properties, such as *in vitro* clonogenic potential and paracrine functions (20–22). In addition, manufacturing strategies for MSCs vary widely, and culture conditions or CPPs can have a marked impact on cell behaviour (23, 24). For example, stimulation with pro-inflammatory cytokines, commonly referred to as “licensing” in the MSC field, has been extensively explored as a means to enhance immunoregulatory functions of MSCs and reduce donor heterogeneity (25, 26).

In terms of CPPs, we elected to focus on and compare MSCs cultured under hypoxic or 3D aggregate conditions as non-genetic, culture manipulating methods that are known to augment immunomodulatory and/or angiogenic properties of MSCs, rather than traditional parameters (medium, seeding density, etc.). The effects of hypoxic conditions on MSC function have been widely investigated, in particular for augmenting the pro-angiogenic functions of MSCs within *in vitro* and *in vivo* models (27, 28). Recent evidence has also shown that hypoxic culture may augment MSC immunomodulatory functions (29, 30). In parallel, 3D cultures of MSCs were considered as work by Bartosh et al. demonstrated that human MSC(M) spheroids had improved immunomodulatory functions within an *in vitro* mouse macrophage co-culture system and in the zymosan-induced peritonitis mouse model (31). Other studies have provided further evidence for the augmented immunomodulatory (32, 33), as well as pro-angiogenic (32, 34, 35) functions of MSCs cultured in 3D aggregates. Notably, we employed xeno-free cell culture medium for MSC expansion and generation of 3D aggregates, and previous work has suggested that 3D-cultured MSCs lose their augmented immunomodulatory function when cultured using xeno-free medium (36).

In the present study, we explored the relationship between select CPPs known to enhance immunomodulatory and/or angiogenic MSC basal fitness range and multivariate morphological, gene expression, soluble factor expression, and functional readouts against a backdrop of donor heterogeneity. Using adipose tissue-derived MSCs (MSC(AT)), we employed a statistical approach to identify a putative matrix of CQAs that are correlated with anchor functional assays. We focused on *in vitro* MΦ polarization, recognizing that MΦs are a primary effector cell type of MSCs for numerous indications (reviewed in (37)), and given clinical data from our laboratory demonstrating that MSC(M) injections modulate MΦ phenotype in knee osteoarthritis (8). To evaluate functional angiogenesis, the human umbilical vein endothelial cell (HUVEC) tube formation assay was selected as a potency assay that has been employed for clinical-grade MSC products (19, 38).

We integrated our statistical methods to identify a matrix of putative CQAs with minimum and maximum values that correlated with functional immunomodulatory and/or angiogenic readouts. This combinatorial assay matrix approach allowed us to systematically compare and rank the effects of varying CPPs on putative CQAs for MSC(AT) in terms of their immunomodulatory and angiogenic properties, two functional axes with high therapeutic relevance. The matrix of putative CQAs also allows for identification of donors with enhanced immunomodulation or angiogenic functionalities.

## Methods

### MSC(AT) isolation, culture, and CPPs

Subcutaneous human adipose tissue was obtained external to the knee joint in patients undergoing knee arthroscopy or from abdominal lipoaspirate (REB #18-5480 and #18-6345, see **Table 1** for summary of donor characteristics). MSC(AT) were isolated and expanded using MesenCult™-ACF Plus xeno-free and antibiotic-free growth medium (StemCell Technologies, Vancouver, Canada) on standard tissue culture polystyrene flasks coated with animal component-free cell attachment substrate (StemCell Technologies) according to the manufacturer’s instructions. Cells were expanded in a standard incubator at 37˚C in 5% CO_2_ under ambient air. For passaging, flasks at approximately 80% confluency were washed with PBS (Wisent, St-Bruno, Canada) and harvested using TrpLE (Gibco, Waltham, USA) prior to re-plating at 5,000 cells/cm^2^. All experiments were performed using MSC(AT) between passage 3 and passage 5.

**Table 1:** Summary of MSC(AT) donor characteristics.

Donor ID	Sex	Age	BMI	Depot	Procedure	Osteoarthritis location	KL grade
D1	M	46	27.3	Knee	Arthroscopy	None	N/A
D2	F	38	22.0	Knee	Arthroscopy	Knee	2
D3	M	28	26.9	Knee	Arthroscopy	Knee	1
D4	F	52	22.4	Knee	Arthroscopy	Knee	1
D5	M	54	30.1	Abdomen	Lipoaspirate	Hand	N/A

Subcutaneous adipose tissue was collected from human donors. Depot column indicates anatomical location of adipose tissue collection. Procedure column indicates the procedure the donor underwent for the adipose tissue collection. All patients (except D1) had been diagnosed with knee or hand osteoarthritis. M: Male, F: Female, BMI: Body Mass Index, KL: Kellgren-Lawrence grade.

At approximately 70-80% confluence, MSC(AT) were transiently (16-20 h) cultured under varied CPP conditions, including 3D normoxic (3D-N), 2D hypoxic (2D-H), or 2D normoxic (2D-N) conditions. These culture steps were performed on separate flasks cultured in parallel using MesenCult™-ACF Plus medium supplemented with 1% (v/v) human serum album (HSA; Canadian Blood Services, Ottawa, Canada). 3D-N culture was performed by harvesting MSC(AT) from adherent flasks and plating cells on ultra-low attachment surfaces (Corning, Corning, USA) at 26,700 cells/cm^2^ and 200,000 cells/mL in medium supplemented with 2 ng/mL IL-6 (Peprotech, Cranbury, USA; used to support cell viability in the 3D culture), based on previously reported methods that allow spontaneous aggregation of MSCs into cell clusters (39). Flasks from the same batch of cells were cultured in parallel under 2D-N (maintained in standard tissue culture incubator) or 2D-H conditions (38 mmHg O_2_, *i.e*., 5% O_2_ under standard atmospheric pressure) using a HypoxyLab workstation (Oxford Optronix, Milton, UK) for the same duration as 3D culture.

Prior to experiments, 3D cell aggregates were collected from ultra-low attachment flasks. Following multiple PBS washes of the flask and mixing, samples were removed for cell counting. Cell enumeration was performed after dissociating the aggregates using Accumax™ solution (Sigma, St. Louis, USA), according to previously published methods (40). For MSC(AT) cultured under 2D-N and 2D-H conditions, flasks were washed with PBS and incubated in TrpLE solution, followed by neutralization with complete medium and cell counting. All cell counts were performed using the Vi-Cell XR Cell Counter (Beckman Coulter, Brea, USA). Prior to plating cells for experiments, excess PBS was added to cell suspensions to dilute residual growth factors/cytokines before centrifugation (350 x g, 5 min) to pellet the cells.

### Morphometric and surface marker characterization of MSC(AT)

Cell diameter and circularity was measured for single cell suspensions using the Vi-Cell XR Cell Counter (Beckman Coulter). 3D-N MSC(AT) were dissociated into single cell suspensions as described above. To analyze maximum feret diameters and circularity of whole intact 3D-N MSC(AT) aggregates, 10X phase-contrast images were captured using an EVOS XL Core Cell Imaging System (ThermoFisher, Waltham, USA). A semi-automated algorithm was developed in ImageJ (41) based on rolling ball subtraction to create binary images for particle analysis, and a minimum of 230 aggregate measurements were performed per donor and condition.

Surface marker expression of MSC(AT) was measured following previously established protocols (8) and in accordance with IFATS/ISCT guidelines (positive marker threshold: >80%, negative marker threshold: <2%) (42). The following PE-conjugated anti-human antibodies from BioLegend (San Diego, USA) were used: anti-CD90 (cat. 328109), anti-CD73 (cat. 344004), anti-CD44 (cat. 338807), anti-CD29 (cat. 303003), anti-CD13 (cat. 301703), anti-CD34 (cat. 343506), anti-CD31 (cat. 303105), anti-CD45 (cat. 304008), and anti-CD105 (cat. 323205). For staining, single cell suspensions of 2D-H and 2D-N MSC(AT) were obtained by TrpLE dissociation, while 3D-N aggregates were digested using Accumax™ solution as described above. To evaluate the effects of Accumax™ digestion on the surface marker profile of 2D MSC(AT), a subset of 2D-N MSC(AT) were digested using the same Accumax™ digestion protocol as used for 3D cell aggregates. Samples were characterized using the FC500 flow cytometer (Beckman Coulter) and analyzed using FlowJo version 10 software (Ashland, USA).

### Western blotting

Western blot analysis was performed to confirm CD105 expression in MSC(AT) cultured under varying CPP conditions. Cells were lysed using a buffer containing 50 mM Tris–HCl (pH 7.5), 1mM EGTA, 1mM EDTA, 1% (w/v) Nonidet P40, 1mM sodium orthovanadate, 50 mM sodium fluoride, 5 mM sodium pyrophosphate, 0.27 M sucrose, and a protease inhibitor cocktail (Roche). Protein concentration of all samples was measured using the Pierce BCA protein assay (ThermoFisher). Protein samples were loaded in a 10% polyacrylamide gel (20 μg/well) for electrophoresis followed by transfer to nitrocellulose membranes. Membranes were then blocked with TBS-T containing 5% (w/v) BSA and they were immunoblotted in the same buffer overnight at 4°C with an anti-CD105 primary antibody (cat. 323205, BioLegend, 1:1,000 dilution), or for 2 h with an anti-β-actin antibody (Sigma, 1:10,000 dilution) used for the loading control. Washes were then performed with TBS-T and the blots were then incubated with secondary HRP-conjugated antibodies in 5% skimmed milk. The blots were washed in TBS-T and the signal was detected with the enhanced chemiluminescence reagent (ECL; GE Healthcare, Chicago, USA) and using a chemiluminescent imaging system (Bio-Rad, Hercules, USA).

### Gene expression and soluble factor measurements

After harvesting cells from 2D-N, 2D-H, and 3D-N conditions, cells were added to 24-well plates at 60,000 cells/well in MesenCult™-ACF Plus medium supplemented with 1% HSA (v/v) with or without addition of pro-inflammatory licensing cytokines. The licensing cytokines (all purchased from Peprotech) consisted of IFNγ (30 ng/mL), TNFα (10 ng/mL), and IL-1β (5 ng/mL). Cells harvested from 2D-N or 2D-H conditions were maintained under normoxic or hypoxic (38 mmHg O_2_) conditions for 24 h, respectively, while cell aggregates from the 3D-N condition were plated on ultra-low attachment 24-well plates (Corning) without dissociation. After the culture period, conditioned medium was collected, centrifuged (1,000 x g, 5 min) and frozen at -80°C. The remaining cells were washed in PBS and RNA was extracted using a RNeasy Mini kit (Qiagen, Hilden, Germany) according to the manufacturer’s instructions. RNA concentration and purity was measured using a DS-11 Spectrophotometer (DeNovix, Wilmington, USA).

The nCounter platform (NanoString, Seattle, USA) was used as a highly sensitive tool that detects target mRNAs with high specificity and without amplification. Samples (100 ng RNA/sample) were run on the nCounter MAX Analysis system (St. Michael’s Genomics Molecular Biology Core facility) according to the manufacturer’s instructions using a custom CodeSet 58-gene panel. The data was processed using the nSolver version 4.0 software (NanoString) according to the manufacturer’s instructions to obtain mRNA counts normalized to the synthetic positive control probes and to reference genes. The following were measured as potential reference genes: *ABCF1*, *GAPDH*, *GUSB*, *HPRT1*, *LDHA*, *RPL19*, *RPLP0*, *TUBB*, *POLR1B*, and *TBP*. Reference gene stability was evaluated using geNorm analysis in the nSolver software; *POLR1B* and *TBP* were subsequently discarded as reference genes and not used for normalization. Normalized mRNA counts below 20 were assigned values of 1 if >33% of samples were within range. The following genes were undetectable (<33% of samples were within range) under both licensed and unlicensed conditions: *ANGPT2*, *BDNF*, *BMP7*, *CCR7*, *CD200*, *CTLA4*, *CXCR4*, *IGF1*, *IL10*, *IL12A*, *NGF*, *PDGFB*, *PROK1*, and *CXCL12*. Under licensed conditions, *VASH1* and *SOX9* were undetectable. Under unlicensed conditions, *CD274*, *IDO1*, *NFKBIA*, *NOS2*, and *PDGFA* were undetectable. We selected a broad spectrum of MSC(AT) transcripts initially but many of the undetectable genes are not commonly or are inconsistently expressed by MSCs. Other genes may have tissue of origin-dependent or context-dependent expression. All NanoString data has been deposited in NCBI’s Gene Expression Omnibus (43) accessible through GEO Series accession number GSE212368.

Conditioned medium samples were analyzed for soluble factors using a custom 10-analyte LEGENDplex immunoassay (BioLegend) according to the manufacturer’s instructions, and samples were run on a FACSCanto™ II flow cytometer (BD Biosciences, Franklin Lakes, USA). Values below the assay limit of detection were imputed as half of the lower limit of detection if >33% of samples were within range. IL-10, PIGF, and PD-L1 were undetectable in the samples tested. IL-1RA was undetectable in unlicensed samples only.

Pilot gene expression experiments were performed to i) examine gene expression in MSC(AT) cultured using the combination of 3D and hypoxic culture (3D-H), and ii) to evaluate effects of IL-6 treatment on MSC(AT) cultured under 2D-N conditions using the same IL-6 concentration and treatment duration as used for 3D-N conditions. Both sets of pilot experiments were performed under licensed conditions using the methods outlined above. After licensing, RNA was isolated from MSC(AT) by Trizol-chloroform extraction and cDNA was generated using SuperScript™ IV VILO™ Master Mix (Invitrogen, Waltham, USA). qPCR was run using custom primers (**Suppl. Table 1**, Invitrogen) and FastStart Universal SYBR Green Master Mix (Roche, Basel, Switzerland) on a QuantStudio™ 5 system (ThermoFisher). Results were normalized (ΔΔCT) against reference genes (*B2M* and *RPL13A*) and presented as fold-change values relative to 2D-N culture conditions.

### *In vitro* MΦ polarization

Peripheral blood-derived CD14^+^ monocytes were isolated from a leukopak (StemCell Technologies) by Ficoll density gradient separation and selection with CD14^+^ magnetic beads as previously described (44). Cryopreserved monocytes were thawed and plated on 24-well plates at 100,000 cells/well and allowed to acclimate for 48 h in co-culture medium consisting of: 1 mM sodium pyruvate (Gibco), 1% penicillin-streptomycin (Gibco), 10% FBS (Wisent), and 10% low-glucose DMEM (Sigma) in RPMI medium (Gibco). Prior to co-culture, MSC(AT) were harvested from 2D-N, 2D-H, and 3D-N conditions, plated on 0.4 μm transwell inserts at 10,000 cells/insert in a separate 24-well plate, and allowed to attach for 2 h. The inserts were then transferred to MΦ wells and co-cultured for 20-24 h. Transwell inserts containing MSC(AT) were then removed, and lipopolysaccharide was spiked into wells at a final concentration of 2.5 ng/mL. After a 4 h incubation, MΦs (both adherent and in suspension) were collected and stored in Trizol (Roche) at -80°C. Conditioned medium was also collected and stored at -80°C.

Levels of TNFα in conditioned medium were measured by ELISA (R&D Systems, Minneapolis, USA) according to the manufacturer’s instructions. To analyze MΦ gene expression, RNA was isolated by Trizol-chloroform extraction and cDNA was generated using SuperScript™ IV VILO™ Master Mix (Invitrogen). qPCR was run using custom primers (**Suppl. Table 1**, Invitrogen) and FastStart Universal SYBR Green Master Mix (Roche) on a QuantStudio™ 5 system (ThermoFisher). Results were normalized (ΔΔCT) against reference genes (*ACTB*, *B2M*, and *TBP*) and presented as fold-change values relative to MΦ cultured without MSC(AT) (SOLO condition).

### *In vitro* HUVEC tube formation

To prepare conditioned medium for the HUVEC tube formation assay, MSC(AT) were harvested from 2D-N, 2D-H, and 3D-N conditions, plated at 15,000 cells/cm^2^ and 100,000 cells/mL of growth medium (no added HSA or exogenous cytokines), and incubated for 24 h in cell culture incubators. MSC(AT) harvested from all three CPP conditions were maintained under these conditions to prepare conditioned medium. To prepare unconditioned medium controls, growth medium was incubated on a cell culture plate for the same duration. Following the 24 h incubation period, the conditioned medium was collected, centrifuged (1,000 x g, 5 min), and the supernatant was frozen at -80°C for future use in the HUVEC tube formation assay.

Five to seven days prior to the tube formation assay, P4 HUVECs (Cat. CC2519, Lonza, Basel, Switzerland) were thawed and expanded in EGM™-2 (Lonza) according to the manufacturer’s instructions. The HUVEC tube formation assay was performed based on previously published methods (38). 96-well plates were pre-coated with Cultrex Reduced Growth Factor Basement Membrane Extract, PathClear (R&D Systems) according to the manufacturer’s instructions. HUVECs were harvested and plated on the coated plates at 42,500 cells/cm^2^ in conditioned medium. The following medium formulations were used as controls: unconditioned medium, EGM™-2 medium (positive control), and basal medium without addition of growth factors/supplements (negative control). All wells were imaged 6 h after plating the cells using the EVOS XL Core Cell Imaging System (ThermoFisher) at 4X objective. The images were analyzed using the Angiogenesis Analyzer plugin in ImageJ (45).

### Statistical analysis

Plots were created using GraphPad Prism 6.0 (La Jolla, USA) and JMP Pro 14 (Cary, USA) software. All statistical tests are specified in the figure captions. One- and two-way ANOVA, as well as simple linear regression was performed using GraphPad Prism software. Unbiased hierarchical clustering (Ward method), principal component (PC) analysis (default estimation method), and desirability profiling was performed using JMP software. Analysis of differential gene expression was performed in nSolver (NanoString) using single linear regressions for each covariate (*i.e*., gene) with false discovery rate (FDR)-corrected p values calculated using the Benjamini-Yekutieli method (46). Data were considered statistically significant based on a threshold of p<0.05. For functional *in vitro* assays (MΦ polarization and HUVEC tube formation) where PC analysis was performed on the assay readouts, the PC1 score was taken as a ‘composite functional score’, based on previously published methods (14). For linear regression, analysis between functional PC1 scores and MSC(AT) characteristics (genes, soluble factors, and morphometric features), statistically significant (p<0.05) correlations are reported along with correlations with 0.05<p<0.1 which were considered as near-significant. Normal residuals were checked to fulfill assumptions of linear regression, and parametric correlations were performed independently for each individual MSC(AT) characteristic that was measured.

Desirability profiling (47) was used as a tool for multiple response optimization that assigns individual responses a score from zero to one based on the range of data values, with zero representing an undesirable response, and one representing a highly desirable response. Minimization or maximization functions were assigned to each MSC(AT) characteristic (including genes, soluble factors, and morphological features) to indicate whether higher or lower values were desirable, based on outcomes from linear regression analyses with functional PC1 scores. Based on the regression analyses, all statistically significant (p<0.05) and near-significant (p<0.1) MSC(AT) characteristics were included in the desirability analysis. The R^2^ values from the regression analyses were applied as weightings for each MSC(AT) characteristic.

## Results

### An *in vitro* assay matrix of readouts to identify putative CQAs

**Figure 1:**
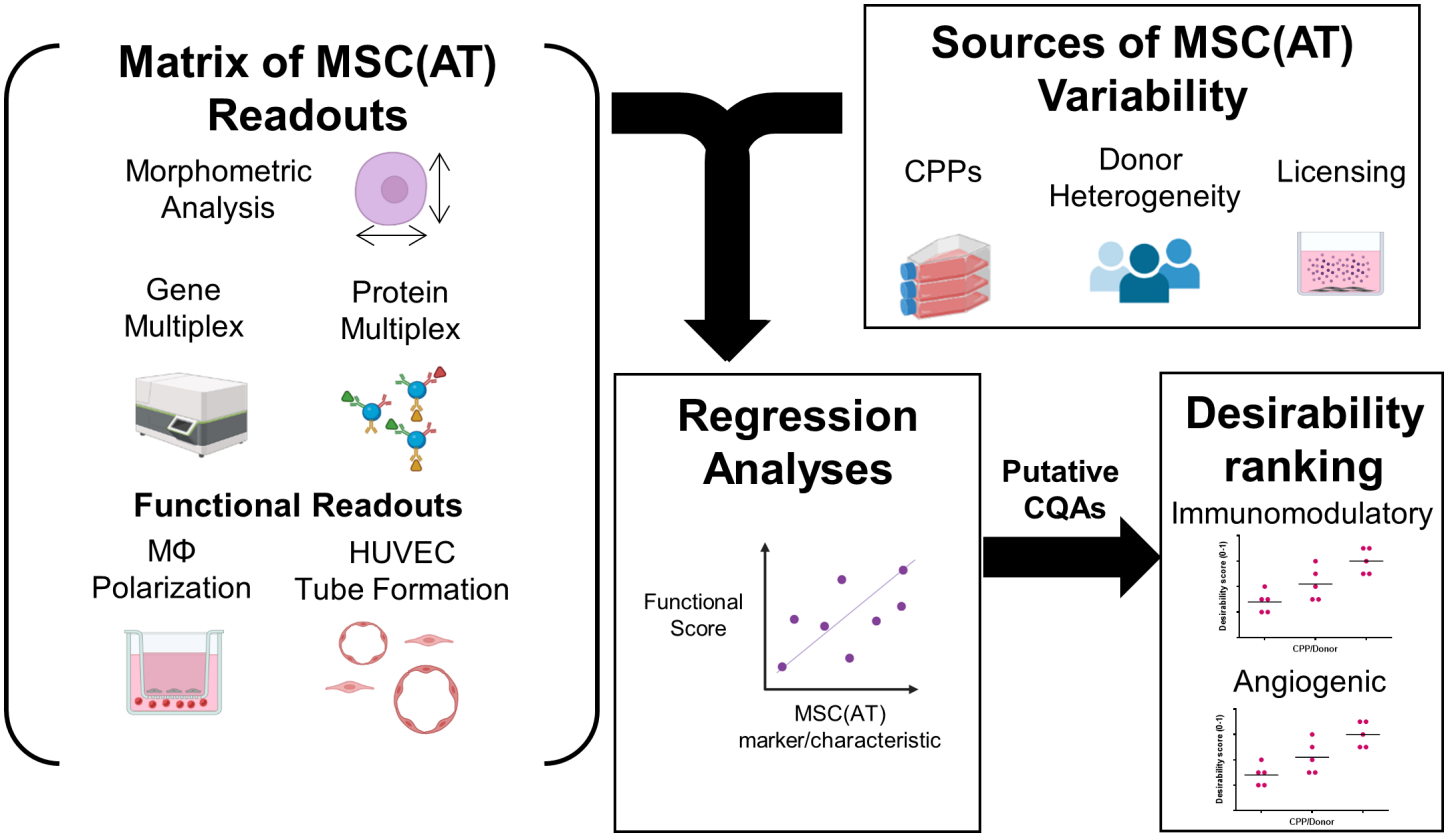
Schematic of experimental and statistical analyses to identify a putative matrix of critical quality attributes sensitive to changes in specific critical processing parameters that enhance MSC(AT) potency. The overarching aim of this study was to evaluate the response of putative critical quality attributes (CQAs) to variations in culture conditions, critical processing parameters (CPPs) selected for their known ability to enhance potency, and donor heterogeneity of MSC(AT). To do this, a matrix of assays consisting of morphometric analysis (of single cell suspensions), gene multiplex (58-gene panel), soluble factor analysis (10-analyte panel), *in vitro* monocyte/macrophage (MΦ) polarization (functional immunomodulatory assay), and *in vitro* human umbilical vein endothelial cell (HUVEC) tube formation assays (functional angiogenic assay) was applied. The matrix of MSC(AT) assays was sensitive to variations in CPPs (including 3D normoxic, 2D hypoxic, and 2D normoxic conditions), donor heterogeneity (N=5 human MSC(AT) donors), and to licensing with pro-inflammatory cytokines. Changes in the matrix of gene and protein expression profiles of MSC(AT), and morphological features correlated with functional immunomodulatory and angiogenic readouts by regression analyses to refine a panel of putative MSC(AT) CQAs. Desirability profiling of these putative CQAs allowed ranking of the effects of CPPs or donor heterogeneity on desired immunomodulatory or angiogenic properties.

A matrix of *in vitro* readouts was used to investigate the responses of putative CQAs to varying donors and CPPs that were specifically selected for their known ability to enhance MSC immunomodulatory and angiogenic properties. The matrix consisted of multivariate morphometric measurements, gene expression, soluble factor analysis, and functional immunomodulatory and angiogenic readouts (**Figure 1**). The matrix of readouts demonstrated sensitivity to multiple sources of MSC(AT) variability, including variations in select CPPs, donor heterogeneity, and licensing with pro-inflammatory cytokines. Linear regression analyses were used to refine putative CQAs by evaluating correlations with anchor functional *in vitro* immunomodulatory and angiogenic outcomes. We further provided a range of values for assessing basal MSC(AT) fitness range according to the significant and near-significant putative CQAs identified from the regression analyses. Desirability analysis was then applied to analyze the profile of putative CQAs and to assign empirical rankings for donors and CPP conditions that result in desirable MSC(AT) immunomodulatory and/or angiogenic functionality.

The CPPs investigated included 2D-N, 2D-H, and 3D-N culture conditions. These CPPs were chosen based on previous literature demonstrating that 3D and 2D hypoxic culture can enhance the immunomodulatory and angiogenic potency of MSCs (28, 29, 31, 32, 35). MSC(AT) cultured under 2D-N, 2D-H and 3D-N conditions satisfied surface marker expression criteria (42) and were CD90^+^CD73^+^CD44^+^CD13^+^CD34^-^CD31^-^CD45^-^ (**Figure S1A**). Notably, CD105 appeared to be cleaved under enzymatic conditions required for dissociating 3D cell aggregates for flow cytometry analysis (**Figure S1B**). Western blot analysis verified CD105 expression by MSC(AT) cultured using 3D-N conditions, albeit at lower levels relative to 2D-N and 2D-H conditions (**Figure S1C**). MSC(AT) cultured under 2D-N and 2D-H conditions displayed a characteristic spindle-like morphology, while MSC(AT) cultured under 3D-N conditions formed cell aggregates of varying sizes (**Figure S2A**). Cell aggregates in the 3D-N condition had a median ferret diameter of 37.82 μm (range: 12.38 – 269.40 μm) and median circularity of 0.56 (range: 0.044 – 0.97). Morphometric measurements of MSC(AT) revealed differences in cell morphology with changes in CPP conditions. Analysis of single cell suspensions obtained from each culture condition demonstrated significantly reduced diameter and greater circularity of 3D-N MSC(AT) relative to 2D-N and 2D-H MSC(AT) (**Figure S2B, C**).

For gene expression and soluble factor analysis, 2D-N, 2D-H, and 3D-N MSC(AT) were subject to pro-inflammatory licensing conditions (with a cocktail of three pro-inflammatory cytokines, TNFα, IFNγ, and IL-1β to simulate a wide range of disease conditions) or cultured under unlicensed conditions in the absence of pro-inflammatory stimuli. The combination of 3D and hypoxic culture (3D-H) was investigated by gene expression analysis using an abbreviated panel of anti-inflammatory/angiogenic markers measured in two MSC(AT) donors (**Figure S3**) and demonstrated no significant benefit of combining these culture conditions. Thus, 3D-N was used as the select CPP in all subsequent experiments. The effects of IL-6 (used to support cell viability under the 3D-N culture condition) were also evaluated on MSC(AT) cultured under 2D-N culture conditions using the same cytokine concentration as used for the 3D-N culture method. An abbreviated panel of genes was selected using markers that were significantly differentially expressed in the 3D-N culture condition relative to 2D-N culture. Gene expression analysis revealed no significant effect of IL-6 on 2D-N culture (**Figure S4**), suggesting that the 3D geometry was the major factor that primed the cells rather than IL-6.

### Curated gene expression and soluble factor profiles are differentially sensitive to donor heterogeneity and select CPPs that enhance MSC potency

**Figure 2:**
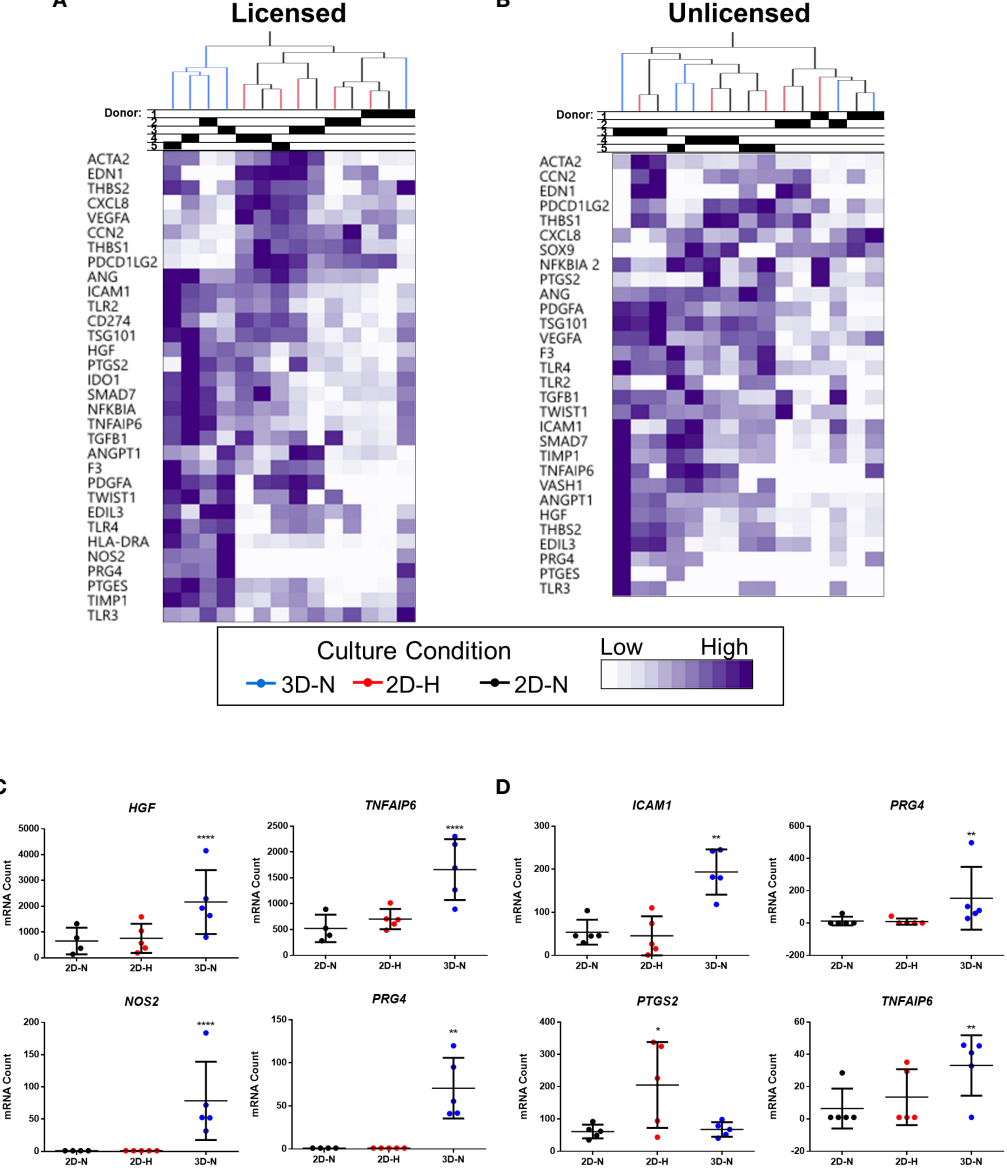
Curated gene expression panel was differentially sensitive to donor heterogeneity and select CPPs that enhance immunomodulatory and/or angiogenic potencies. A and B) Unbiased hierarchical clustering of normalized mRNA counts demonstrated clustering by CPPs (colour-coded dendrogram) and by donor (shaded regions in Donor chart indicate different donors) under both Licensed **(A)** and Unlicensed **(B)** conditions. C and D) Select genes were differentially expressed by modifying CPPs under Licensed **(C)** and Unlicensed **(D)** conditions. Multivariate linear regression, Benjamini-Yekutieli False Discovery Rate-corrected p values. *p<0.05, **p<0.01, ****p<0.0001 vs 2D-N. Horizontal bars: group mean, error bars: standard deviation. 3D-N, 3D Normoxic culture; 2D-N, 2D Normoxic culture; 2D-H, 2D Hypoxic culture. N=5 MSC(AT) donors, n=1 technical replicate due to high sensitivity of Nanostring measurements.

Gene expression analysis was performed using a curated panel of markers including predominantly immunomodulatory and angiogenic genes (data provided in **Suppl. Tables 2, 3, 4, and 5**). The panel was selected based on previous literature (11, 17, 48, 49) and our experience using MSC(M) in an osteoarthritis clinical trial (8). It was used to evaluate MSC(AT) fitness across multiple donors and while modulating select CPP conditions associated with enhanced immunomodulatory and angiogenic functionality. Unbiased hierarchical clustering analysis revealed that MSC(AT) gene expression profiles clustered according to both variations in CPP conditions and donor heterogeneity (**Figures 2A, B**). Under licensed conditions, CPP variations, specifically 3D-N configurations of culturing MSC(AT) clustered separately from 2D-N and 2D-H cultures for all but one donor (Donor 1) which clustered together regardless of CPP variations. For MSC(AT) cultured under 2D-N and 2D-H conditions, the gene expression profiles clustered together by donor rather than oxygen tension under licensed conditions. Interestingly, under unlicensed conditions, gene expression profiles clustered primarily by donor, regardless of variations in CPPs. Overall, variations in CPP conditions by changing culture geometry or oxygen tension and donor heterogeneity resulted in shifts in gene expression profiles dependent on pro-inflammatory licensing conditions or unlicensed conditions. For example, genes such as *HGF* (multifunctional growth factor) and *TNFAIP6* (anti-inflammatory gene encoding TSG6), were upregulated by 3D-N culture conditions relative to 2D-N culture conditions, under licensed conditions (**Figure 2C**). *NOS2* (encoding for inducible nitric oxide synthase, iNOS which may indicate cell stress or enhanced immunosuppressive functions for mouse, but not human MSCs (50)) and *PRG4* (lubricating proteoglycan with potential immunomodulatory functions (51, 52)) were also upregulated under these conditions. *ICAM1* (immunomodulatory marker), *PRG4*, and *TNFAIP6* were upregulated by 3D-N culture conditions relative to 2D-N under unlicensed conditions (**Figure 2D**). Furthermore, 2D-H culture conditions induced augmented expression of the immunomodulatory marker *PTGS2* relative to 2D-N culture conditions under unlicensed settings.

**Figure 3:**
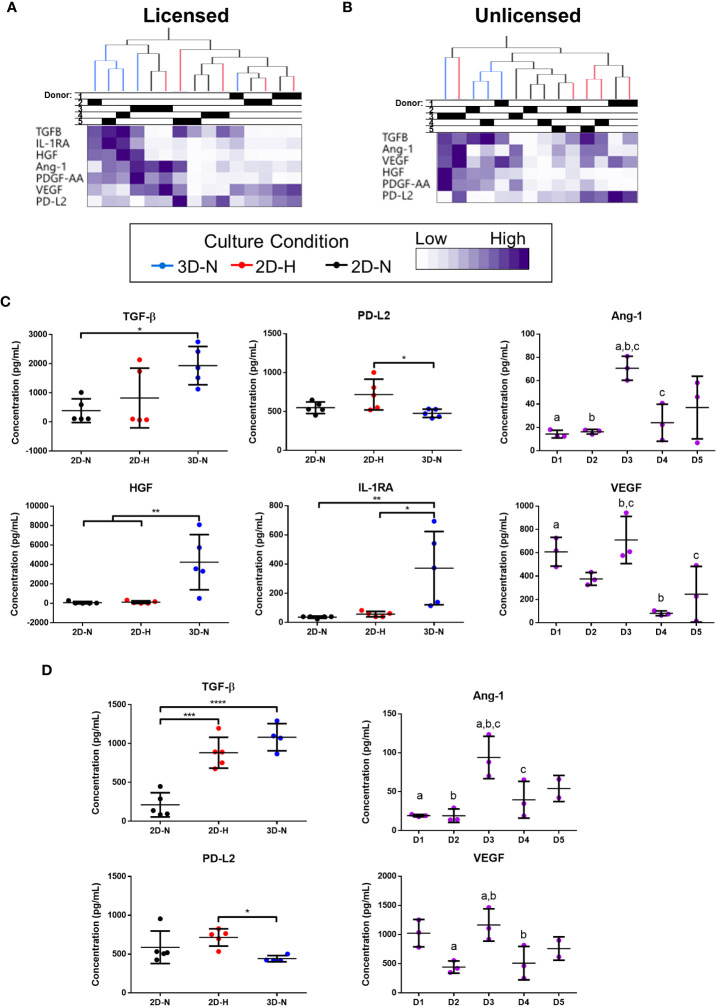
Curated anti-inflammatory and angiogenic soluble factors were differentially sensitive to donor heterogeneity and select CPPs that enhance immunomodulatory and/or angiogenic potencies. **(A, B)** Unbiased hierarchical clustering of soluble factors demonstrated clustering by CPPs and donor under Licensed **(A)** and Unlicensed **(B)** conditions. **(C, D)** Soluble factors with statistically significant differences between CPPs (left) or donors (right) are displayed for Licensed **(C)** and Unlicensed **(D)** conditions. One-way ANOVA, Tukey’s *post-hoc* test. *p<0.05, **p<0.01, ***p<0.001, ****p<0.0001. Donors sharing same letter are significantly different (p<0.05). Horizontal bars: group mean, error bars: standard deviation. Data points represent mean of technical replicates for each donor and condition. 3D-N, 3D Normoxic culture; 2D-N, 2D Normoxic culture; 2D-H, 2D Hypoxic culture. N=5 MSC(AT) donors, n=2 technical replicates.

Levels of soluble factors in conditioned medium were queried using a curated sub-panel of immunomodulatory and angiogenic factors, based on significant results from the gene expression analysis (soluble factor data provided in **Suppl. Tables 6, 7**). Unbiased hierarchical clustering demonstrated that soluble factor profiles clustered based on both donor heterogeneity and variations in CPP conditions under licensed and unlicensed conditions (**Figures 3A, B**). Analysis of individual soluble factors was performed to evaluate the effects of CPP and donor heterogeneity on each soluble factor. Under licensed conditions, MSC(AT) cultured in 3D-N configurations expressed significantly higher levels of the multifunctional cytokine TGF-β relative to 2D-N, and significantly lower levels of the immunomodulatory factor soluble PD-L2 relative to 2D-H (**Figure 3C**). Furthermore, MSC(AT) cultured in 3D-N configurations expressed significantly higher levels of the growth factor HGF and the immunosuppressive factor IL-1RA relative to both 2D-N and 2D-H MSC(AT) under licensed conditions. Under unlicensed conditions, 2D-H and 3D-N MSC(AT) expressed significantly higher levels of TGF-β relative to 2D-N, while soluble PD-L2 was significantly upregulated by 2D-H relative to 3D-N MSC(AT) (**Figure 3D**). Expression of the angiogenic markers angiopoietin-1 (Ang-1) and VEGF showed statistically significant differences between donors, with Donor 3 expressing the highest levels of both factors under both licensed and unlicensed conditions (**Figures 3C, D**). Taken together, the curated panel of genes and soluble factors were differentially sensitive to donor heterogeneity and CPPs under licensed and unlicensed conditions. Interestingly, donor heterogeneity was masked under licensed conditions, and dominated under unlicensed conditions. Further, culturing MSC(AT) under 3D-N conditions rendered them with an elevated profile of anti-inflammatory/immunosuppressive genes (*HGF*, *TNFAIP6*, *PTGES*, *PTGS2*, *TLR2*, *NFKBIA*, *TGFB1*, *PRG4*, *IDO*, *ICAM1*, *TLR4*) and soluble factors (TGF-β, HGF, IL-1RA), corroborating previous reports that have investigated MSC culture in 3D formats (31, 32, 53).

### MSC(AT)-mediated *in vitro* functional polarization of MΦ readouts are dependent on donor heterogeneity and CPPs

**Figure 4:**
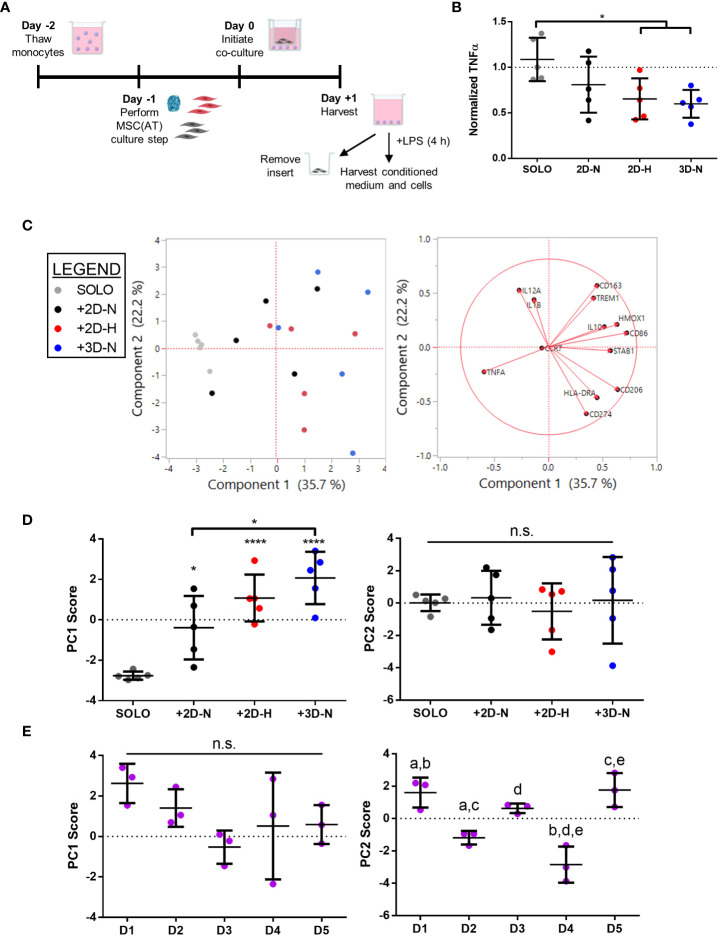
*In vitro* MSC(AT)-mediated functional polarization of MΦ was differentially sensitive to donor heterogeneity and select CPPs. **(A)** Schematic of indirect co-culture experiment. MSC(AT) (2D-N, 2D-H, or 3D-N culture conditions) were co-cultured with human peripheral blood-derived monocytes/macrophages (MΦ) for 24 h prior to removal of MSC(AT) and addition of lipopolysaccharide (LPS). **(B)** TNFα secretion, a surrogate marker for pro-inflammatory MΦs, showed significant reduction when co-cultured with 3D-N or 2D-H MSC(AT) compared to solo MΦs. One-way ANOVA, Tukey *post-hoc* test. *p<0.05. **(C)** Principal component (PC) analysis of median delta-delta Ct values of MΦ genes, indicative of pro-inflammatory (*CCR7*, *CD86*, *HLADRA*, *IL12A*, *IL1B*, *TREM1*) or pro-resolving (*CD163*, *CD206*, *HMOX1*, *IL10*, *CD274*, *STAB1*) status, and normalized TNFα levels from each donor and culture condition (left). The corresponding loading plot of eigenvectors for each marker (right) indicates the relative contribution of each factor to the PCs. **(D)** Principal component scores for PC1 and PC2 according to culture condition. PC1 scores were highest in the 3D-N co-culture condition. One-way ANOVA, Tukey *post-hoc* test. *p<0.05, ****p<0.0001 relative to MΦ SOLO condition or to groups indicated by brackets. **(E)** Principal component scores for PC1 and PC2 according to MSC(AT) donor heterogeneity. Statistically significant differences between groups were observed in PC2 scores and are indicated by groups sharing the same letter. One-way ANOVA, Tukey *post-hoc* test, p<0.01. N=5 MSC(AT) donors, n=3 technical replicates/condition. Horizontal bars: group mean, error bars: standard deviation. 3D-N, 3D Normoxic culture; 2D-N, 2D Normoxic culture; 2D-H, 2D Hypoxic culture; n.s., statistically non-significant.

To further probe immunomodulatory properties of MSC(AT), an indirect co-culture assay was performed to evaluate functional MΦ polarization (**Figure 4A**). Alterations to CPP conditions by changing MSC(AT) culture configuration (3D-N) or oxygen tension (2D-H) significantly reduced levels of pro-inflammatory TNFα in conditioned medium relative to MΦs alone (**Figure 4B**), suggesting that these culture conditions enhanced anti-inflammatory functions of MSC(AT). Gene expression analysis of MΦs revealed statistically significant increased expression of inflammation-resolving MΦ markers (*CD206*, *HMOX1*, and *IL10*) in co-cultures with MSC(AT) in 3D-N and 2D-H culture conditions relative to MΦ alone (**Figure S5A**). In addition, MSC(AT) cultured in 3D-N conditions significantly upregulated MΦ expression of *CD86* (pro-inflammatory) and *CD163* (inflammation-resolving), while 2D-N culture conditions significantly upregulated expression of *HMOX1* only. PC analysis was applied as an unbiased dimension reduction tool to evaluate the full gene expression panel and TNFα protein levels (**Figure 4C**, loading plots for PC1 and PC2 displayed in **Figures S5B, C**). Increased expression of *CD86*, *CD206*, *HMOX1*, and *STAB1* genes, and reduced expression of TNFα protein were the main contributors to higher scores along the PC1 axis (accounting for 35.7% of variation). Reduced expression of *CD274* and *HLADRA*, and increased expression of *CD163*, *IL12A*, and *TREM1* genes drove higher scores along the PC2 axis (accounting for 22.2% of variation).

To further probe the results of the PC analysis, we plotted individual PC1 and PC2 scores for each CPP and donor combination as these scores capture multivariate heterogeneity in a reduced, single dimension with PC1 capturing a larger proportion of the total existing variation in the dataset relative to PC2. Analysis of individual PC1 scores demonstrated that separation along the PC1 axis was driven by co-culture with MSC(AT) as indicated by statistically significant differences in PC1 scores for MSC(AT) cultured under 2D-N, 2D-H, and 3D-N conditions relative to solo MΦ (positive control) without MSC(AT) (**Figure 4D**). Given that the PC1 axis accounted for differences in MΦ phenotypic marker profiles relative to positive control, PC1 scores were considered as a ‘composite functional score’ (14) for MSC(AT) immunomodulatory function. With the exception of *CD86* gene expression, higher scores along the PC1 axis were generally associated with greater inflammation-resolving MΦ polarization as evidenced by increased expression of *CD206*, *HMOX1*, and *STAB1*, along with reduced expression of TNFα protein. Using PC1 scores as an analytic, variations in CPP conditions resulted in differences in MSC(AT)-mediated MΦ polarization toward inflammation-resolving subtypes. Notably, MSC(AT) cultured in 3D-N configurations displayed the highest PC1 scores, and these were significantly different from 2D-N MSC(AT), suggesting that 3D-N MSC(AT) displayed a superior capacity to polarize MΦ phenotype toward inflammation-resolving subtypes. No significant differences in culture conditions were observed along the PC2 axis. Analysis of PC1 scores across individual donors showed no significant differences (**Figure 4E**); while not significant, Donor 1 displayed the highest PC1 scores suggesting that Donor 1 may have intrinsic improved immunomodulatory basal functionality, and Donor 3 displayed the lowest scores. In contrast, a significant effect of donor heterogeneity was observed along the PC2 axis. Given that PC2 scores represent changes in both pro-inflammatory and inflammation-resolving MΦ markers, these data suggest that donor heterogeneity dictates MSC(AT)-mediated polarization of MΦ toward mixed phenotypes.

### *In vitro* MSC(AT)-mediated functional angiogenesis readouts are dependent on donor heterogeneity and CPPs

**Figure 5:**
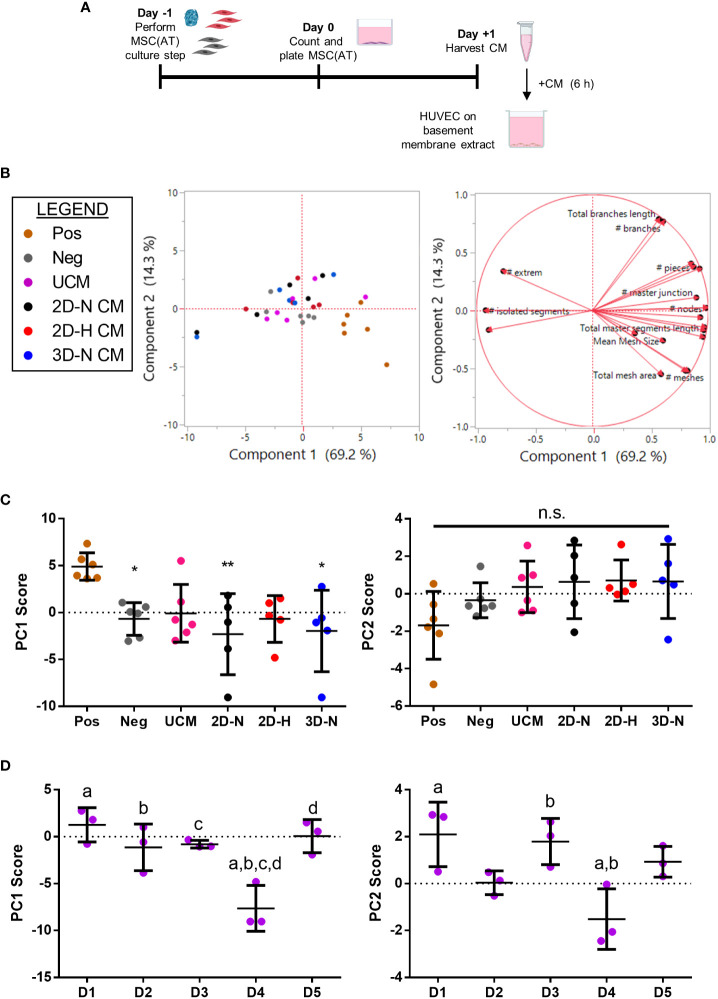
*In vitro* MSC(AT) mediated functional angiogenic HUVEC tube formation assay was differentially sensitive to donor heterogeneity and CPPs. **(A)** Schematic of experimental set-up for the tube formation assay. Conditioned medium (CM) was collected from MSC(AT) (2D-N, 2D-H, or 3D-N) after 24 h of incubation and added to HUVECs cultured on basement membrane extract for 6 h. **(B)** Principal component (PC) analysis of fold-change values (relative to negative control) of twenty HUVEC tube formation image analysis readouts (left). The corresponding loading plot of eigenvectors (right) indicates the relative contribution of each factor to the PCs. **(C)** Principal component scores for PC1 and PC2 according to culture condition. The positive control group (Pos; HUVECs cultured in pro-angiogenic medium) displayed significantly higher PC1 scores relative to the negative control (Neg; HUVECs cultured in basal medium), and to HUVECs cultured with conditioned medium derived from 2D-N or 3D-N MSC(AT) culture conditions. One-way ANOVA, Tukey *post-hoc* test. *p<0.05, **p<0.01 relative to positive control. **(D)** Principal component scores for PC1 and PC2 according to MSC(AT) donor heterogeneity. Statistically significant differences between donors were observed for PC1 and PC2 scores and are indicated by donors sharing the same letter. One-way ANOVA, Tukey *post-hoc* test, p<0.05. N=5 MSC(AT) donors, n=2-3 technical replicates/condition. Horizontal bars: group mean, error bars: standard deviation. HUVEC, human umbilical vein endothelial cell; Pos, positive control; Neg, negative control; UCM, unconditioned medium; 3D-N, 3D Normoxic culture; 2D-N, 2D Normoxic culture; 2D-H, 2D Hypoxic culture; CM, conditioned medium; n.s., statistically non-significant.

To evaluate the angiogenic functions of MSC(AT), the effects of MSC(AT) conditioned medium on HUVEC tube formation was explored (**Figure 5A**, **Figure S6A**). Recognizing that there is no standard quantitation method for HUVEC tube formation and that different measurements can yield different insights into *in vitro* angiogenesis (54), we employed the ImageJ Angiogenesis Analyzer plugin which provides twenty different types of measurements (45). PC analysis was applied to analyze all twenty parameters that profile tube formation image analyses (**Figure 5B**, loading plots for PC1 and PC2 displayed in **Figure S6B, C**). An increased number of nodes, number of junctions, and the total master segment length were the main contributors to higher scores along the PC1 axis (accounting for 69.2% of variation), indicative of greater angiogenesis. Increased total branches length and number of branches, along with reduced total mesh area drove higher scores along the PC2 axis (accounting for 14.3% of variation). Analysis of individual PC1 scores demonstrated that the positive control group (HUVECs cultured in pro-angiogenic medium) displayed significantly higher PC1 scores relative to the negative control (HUVECs cultured in basal medium), and to HUVECs cultured in conditioned medium derived from 2D-N or 3D-N MSC(AT) culture conditions (**Figure 5C**). As above, the PC1 scores were considered as a ‘composite functional score’ for MSC(AT) angiogenic functions given that higher scores along this axis reflected greater HUVEC tube formation. Surprisingly, conditioned medium derived from MSC(AT) cultured under 2D-H or 3D-N conditions did not elicit significant increases in HUVEC PC1 scores, and there were no significant differences between PC1 scores across MSC(AT) by varying CPP conditions. PC1 scores were instead driven by MSC(AT) donor differences (**Figure 5D**). Donors 1, 2, 3, and 5 all displayed statistically significant higher scores relative to Donor 4 indicative of greater angiogenic function. Analysis of PC2 scores similarly showed no significant effect of the conditioned medium from the different groups representing variability in culture conditions, or the controls on HUVEC tube formation profiles (**Figure 5C**). Some variation along the PC2 axis was driven by donor with Donors 1 and 3 displaying significantly higher PC2 scores relative to Donor 4 (**Figure 5D**).

To further analyze effects mediated by variations in donor and experimental batches, PC analysis was performed on *all* biological and technical replicates (**Figure S7A**) and PC1 scores were investigated for each donor (**Figures S7B, C**). Under this analysis, conditioned medium derived from MSC(AT) cultured under 2D-H conditions had the highest mean HUVEC PC1 scores for four out of five donors (Donor 2, 3, 4, and 5) compared to scores for the 2D-N and 3D-N conditions. Taken together, the data suggests that donor heterogeneity dominated over effects mediated by variations in CPPs in our analysis of angiogenic readouts. The net effect of CPPs on HUVEC tube formation was variable, donor-dependent, and in part driven by CPP conditions that favour angiogenesis.

### Generating a matrix of putative MSC(AT) CQAs based on correlation of select genes, soluble factors, and morphological features with functional immunomodulation

**Figure 6:**
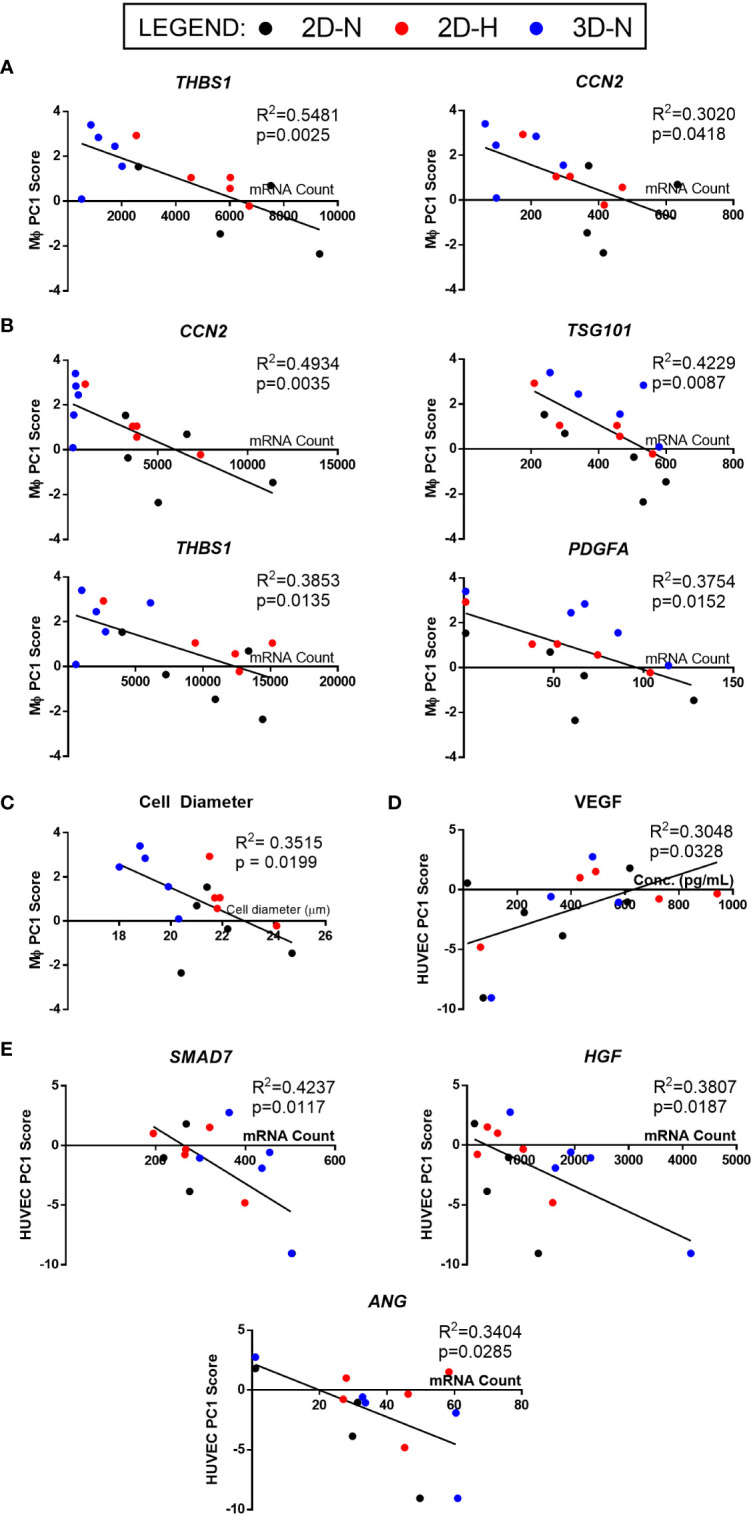
Linear regression analysis revealed genes, soluble factors, and morphological features that correlate with anchor functional assays to identify a putative matrix of CQAs for MSC(AT). **(A, B)** Inverse correlation between PC1 composite scores from MΦ polarization (**Figure 4**) and curated panels of MSC(AT) genes measured under Licensed **(A)** and Unlicensed **(B)** conditions. **(C)** Single cell diameter of MSC(AT) inversely correlated to MΦ polarization PC1 composite scores. **(D)** Positive correlation between HUVEC tube formation PC1 composite scores (**Figure 5**) and levels of VEGF protein in MSC(AT) conditioned medium under Licensed conditions. **(E)** Inverse correlation between HUVEC tube formation PC1 scores and curated panels of MSC(AT) genes measured under Licensed conditions. Only correlations with R^2^≥0.3 are shown. N=14-15. 3D-N, 3D Normoxic culture; 2D-N, 2D Normoxic culture; 2D-H, 2D Hypoxic culture.

The full set of gene and soluble factor expression profiles as well as morphological features measured for MSC(AT) were examined for correlations to composite functional (PC1) scores (indicative of inflammation-resolving MΦ polarization) generated for MSC(AT) immunomodulatory fitness. Under licensed conditions, MSC(AT) expression of *THBS1* (R^2^ = 0.5481, p=0.0025, angiogenic gene), *CCN2* (R^2^ = 0.3020, p=0.0418, encoding for the multifunctional growth factor), and *EDN1* (R^2^ = 0.2854, p=0.0491, angiogenic gene) genes demonstrated significant ***inverse*** correlations with MΦ PC1 composite scores (**Figure 6A**, **Table 2**), suggesting that lower expression of these genes correlated with improved immunomodulatory MSC(AT) functionality. Correlations of MΦ PC1 composite scores with *ACTA2*, *PDCD1LG2*, *TNFAIP6*, *ANGPT1*, and *CXCL8* were near-significant. Under unlicensed conditions, MSC(AT) expression of six genes (*CCN2*, *TSG101*, *THBS1*, *PDGFA*, *VEGFA*, and *EDIL3*) were significantly inversely correlated with MΦ PC1 composite scores, with *CCN2* (R^2^ = 0.4934, p=0.0035), *TSG101* (R^2^ = 0.4229, p=0.0087, negative growth regulator and regulator of vesicular trafficking), *THBS1* (R^2^ = 0.3853, p=0.0135), and *PDGFA* (R^2^ = 0.3754, p=0.0152) displaying the strongest correlations (**Figure 6B**, **Table 2**). Correlations of MΦ PC1 composite scores with *ACTA2*, TGF-β protein, *ANGPT1*, and *ANG* were near-significant. Interestingly, many of the statistically significant and near-significant correlations were inverse correlations and they predominantly consisted of angiogenic-associated genes (*THBS1, CCN2, EDN1, PDGFA*, *VEGFA*, *EDIL3*, *ANGPT1*, and *ANG*).

**Table 2:** Putative CQAs for MSC(AT) immunomodulatory fitness.

Marker/Characteristic	Licensed/Unlicensed	Gene/protein	Min/Max Function	Min value	Max value	p value	R^2^ value
*THBS1*	Licensed	Gene	Min	296.30	1680.04	*0.0025	0.5481
*CCN2*	Unlicensed	Gene	Min	289.15	11393.39	*0.0035	0.4934
*TSG101*	Unlicensed	Gene	Min	209.22	599.97	*0.0087	0.4229
*THBS1*	Unlicensed	Gene	Min	575.38	15145.48	*0.0135	0.3853
*PDGFA*	Unlicensed	Gene	Min	1.00	127.95	*0.0152	0.3754
Cell Diameter	N/A	N/A	Min	18.00	24.70	*0.0199	0.3515
*CCN2*	Licensed	Gene	Min	63.40	634.06	*0.0418	0.302
*VEGFA*	Unlicensed	Gene	Min	214.27	894.31	*0.0378	0.2914
*EDN1*	Licensed	Gene	Min	1.00	65.27	*0.0491	0.2854
*EDIL3*	Unlicensed	Gene	Min	31.41	887.90	*0.0477	0.2689
*ACTA2*	Licensed	Gene	Min	54.88	201.10	0.0614	0.2619
*PDCD1LG2*	Licensed	Gene	Min	47.65	329.96	0.0628	0.2595
*TNFAIP6*	Licensed	Gene	Max	284.00	2300.07	0.0742	0.2417
*ACTA2*	Unlicensed	Gene	Min	78.54	3197.28	0.0736	0.2256
TGFβ	Unlicensed	Protein	Max	85.54	1292.81	0.0865	0.2251
*ANGPT1*	Licensed	Gene	Min	42.27	237.48	0.0868	0.2247
*ANGPT1*	Unlicensed	Gene	Min	27.79	327.12	0.0749	0.2238
*CXCL8*	Licensed	Gene	Min	25008.42	51553.32	0.0946	0.2154
*ANG*	Unlicensed	Gene	Min	25.10	88.99	0.0861	0.2096
Cell Circularity	N/A	N/A	Max	0.47	0.77	0.0981	0.1964

Summary of regression analyses between MΦ PC1 composite scores and MSC(AT) genes, soluble factors, and morphological features. Marker/Characteristic column indicates the gene or protein symbol, or morphological feature. Licensed/Unlicensed column indicates whether the factor was measured under licensed or unlicensed conditions. Min/Max Function column indicates whether minimization or maximation of the MSC(AT) characteristic was desirable based on positive or negative correlations in the linear regression analysis. Min value and Max value columns indicate the range of values measured across each gene (units: mRNA count), soluble factor (units: pg/mL), and morphological feature (units for cell diameter: microns, units for cell circularity: arbitrary). All significant (*p<0.05) and near-significant (p<0.1) correlations are displayed.

Analysis of morphological features of MSC(AT) single cell suspensions derived under varying CPP conditions and/or donors revealed that cell diameter was significantly inversely correlated with inflammation-resolving MΦ PC1 composite scores (**Figure 6C**, **Table 2**), suggesting that smaller cells exhibit greater immunomodulatory properties, concordant with observations by Klinker *et al.* (15). Correlation of MΦ PC1 composite scores with cell circularity was near-significant (**Table 2**). Taken together, the significant and near-significant correlations between MSC(AT) gene expression and morphometric features with MΦ pro-resolving polarization constituted a matrix of multivariate readouts that were considered as putative CQAs for informing MSC(AT) immunomodulatory fitness.

### Generating a matrix of putative MSC(AT) CQAs based on correlation of select genes, soluble factors, and morphological features with functional angiogenesis

Linear regression analyses were also performed to investigate correlations of MSC(AT) markers with functional angiogenic HUVEC PC1 composite scores. A statistically significant ***positive*** correlation was found between HUVEC PC1 composite scores and levels of the pro-angiogenic factor VEGF in MSC(AT) conditioned medium measured under licensed conditions (R^2^ = 0.3048, p=0.0328) (**Figure 6D**), while VEGF levels measured under unlicensed conditions showed a positive near-significant correlation (R^2^ = 0.2416, p=0.0743) (**Table 3**). No significant or near-significant correlations to HUVEC PC1 scores were observed for the other measured soluble factors. Regression analyses of HUVEC PC1 scores with MSC(AT) genes revealed significant ***inverse*** correlations for *SMAD7* (R^2^ = 0.4237, p=0.0117, inhibitor of TGF-β signaling), *HGF* (R^2^ = 0.3807, p=0.0187), and *ANG* (R^2^ = 0.3404, p=0.0285, pro-angiogenic marker) measured under licensed conditions (**Figure 6E**). Correlations of HUVEC PC1 composite scores with expression of *IDO1*, *TSG101*, and *CXCL8* were near-significant (**Table 3**). Under unlicensed conditions, expression of the chondrogenic marker *SOX9* (R^2^ = 0. 2906, p=0.0381) was significantly ***inversely*** correlated with HUVEC PC1 scores (**Table 3**). Inverse correlations of HUVEC PC1 composite scores with expression of *TNFAIP6* were near-significant. No significant or near-significant correlations were observed between MSC(AT) cell diameter and circularity measurements with HUVEC PC1 composite scores. As above, the significant and near-significant correlations between expression levels of VEGF protein and the identified genes with HUVEC tube formation were considered as a matrix of multivariate readouts that served as putative CQAs for informing MSC(AT) angiogenic fitness.

**Table 3:** Putative CQAs for MSC(AT) angiogenic fitness.

Marker/Characteristic	Licensed/Unlicensed	Gene/protein	Min/Max Function	Min value	Max value	p value	R^2^ value
*SMAD7*	Licensed	Gene	Min	194.72	504.28	*0.0117	0.4237
*HGF*	Licensed	Gene	Min	139.8	4151.35	*0.0187	0.3807
*ANG*	Licensed	Gene	Min	1	61.01	*0.0285	0.3404
VEGF	Licensed	Protein	Max	15.2	942.15	*0.0328	0.3048
*SOX9*	Unlicensed	Gene	Min	1	61.46	*0.0381	0.2906
*IDO1*	Licensed	Gene	Min	4326.67	17824.17	0.0501	0.2832
VEGF	Unlicensed	Protein	Max	250.575	1463.65	0.0743	0.2416
*TSG101*	Licensed	Gene	Min	272.06	488.06	0.0744	0.2413
*CXCL8*	Licensed	Gene	Min	25008.42	51553.32	0.0879	0.2234
*TNFAIP6*	Unlicensed	Gene	Min	1	45.68	0.0809	0.216

Summary of regression analyses between HUVEC PC1 composite scores and MSC(AT) genes, soluble factors, and morphological features. Marker/Characteristic column indicates the gene or protein symbol, or morphological feature. Licensed/Unlicensed column indicates whether the factor was measured under licensed or unlicensed conditions. Min/Max Function column indicates whether minimization or maximation of the MSC(AT) characteristic was desirable based on positive or negative correlations in the linear regression analysis. Min value and Max value columns indicate the range of values measured across each gene (units: mRNA count) and soluble factor (units: pg/mL). All significant (*p<0.05) and near-significant correlations (p<0.01) are displayed.

### Statistical rankings of the matrix of putative CQAs ranks different CPPs and donors for optimal MSC(AT) immunomodulation and angiogenic fitness

Desirability profiling was applied as an analytical tool using the matrix of putative CQAs to empirically rank CPP conditions and MSC(AT) donors that favour immunomodulation or angiogenic fitness. Putative CQAs were selected using a broader p value threshold of p<0.1 (near-significance) based on the regression analyses presented above. This p value threshold was selected to filter the large initial panels of genes and soluble factors (curated based on literature) through a pipeline with a still flexible threshold that allowed selection of a relatively broad array of putative CQAs for MSC(AT) immunomodulatory or angiogenic basal fitness. The genes and soluble factors (measured under either licensed or unlicensed conditions), as well as morphological features were assigned minimization or maximization functions in the desirability analysis based on whether the correlation to functional outcomes was positive or negative (**Tables 2** and **3**). For example, markers with negative correlations to inflammation-resolving MΦ or pro-angiogenic HUVEC PC1 composite scores (*i.e*., lower expression of the marker correlated to greater immunomodulatory/angiogenic function) were assigned minimization functions so that lower expression of the marker corresponded to a higher immunomodulatory or angiogenic desirability score. Furthermore, the R^2^ values were used as indicators of the amount of variation in the data explained by the model for each gene/soluble factor/morphological feature in correlation with either immunomodulatory or angiogenic functional outcomes. Thus, these values were applied as individual weightings for each gene, soluble factor, or morphological feature. For example, expression of *THBS1* by licensed MSC(AT) correlated most strongly with inflammation-resolving MΦ PC1 composite scores (R^2^ = 0.5481); the R^2^ value was used as a relative weighting such that *THBS1* expression levels contributed more strongly to the overall desirability score relative to the other genes, soluble factors, and morphological features included in the analysis. Given that putative CQAs would require limits or ranges in order to be practically used, we provided a range of data outputs for each MSC(AT) readout (gene, soluble factor, and morphological feature) that correlated with immunomodulatory pro-resolving MΦ polarization or HUVEC tube formation (**Tables 2** and **3**). These values inform the upper and lower limits of the putative CQA matrix readouts corresponding to MSC(AT) immunomodulation (**Table 2**) or angiogenic fitness (**Table 3**).

**Figure 7:**
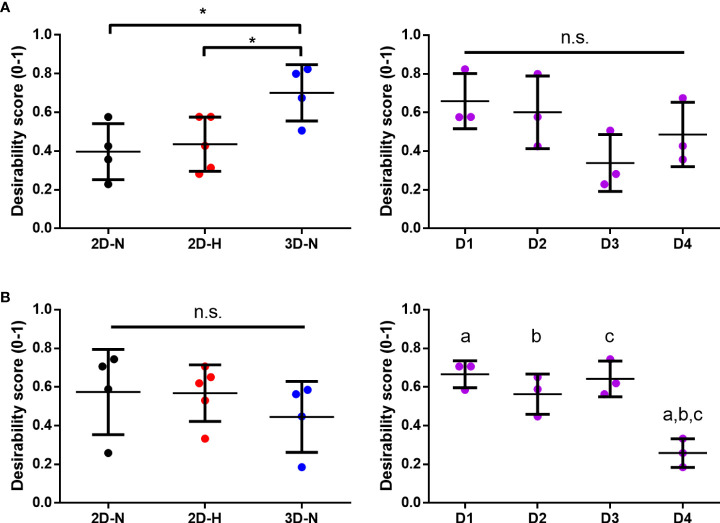
Statistical rankings of matrix of putative CQAs ranked different CPP conditions known to enhance MSC potency, and donors for optimal immunomodulation and angiogenic functionalities. Desirability analysis was performed on putative CQAs selected for evaluation of immunomodulatory **(A)** and angiogenic **(B)** functionalities. **(A)** Desirability profiling ranked 3D-N CPP conditions higher than 2D-H and 2D-N for overall immunomodulatory desirability scores, while scores were similar across donors. **(B)** Desirability profiling ranked Donors 1, 2, and 3 higher than Donor 4 for overall angiogenic desirability scores, while scores were similar across various CPPs. One-way ANOVA, Tukey *post-hoc* test. *p<0.05. Donors sharing same letter indicate statistically significant differences (p<0.05). Horizontal bars: group mean, error bars: standard deviation.  3D-N, 3D Normoxic culture; 2D-N, 2D Normoxic culture; 2D-H, 2D Hypoxic culture, n.s, non-significant.

Using the panels of MSC(AT) genes, soluble factors, and morphological features that correlated with inflammation-resolving MΦ PC1 scores (Licensed panel: *ACTA2*, *ANGPT1*, *CCN2*, *CXCL8*, *EDN1*, *PDCD1LG2*, *THBS1*, *TNFAIP6*; Unlicensed panel: *ACTA2*, *ANG*, *ANGPT1*, *CCN2*, *EDIL3*, *PDGFA*, TGF-β, *THBS1*, *TSG101*, *VEGFA*; Morphological features: cell diameter, cell circularity), desirability analysis revealed that MSC(AT) cultured under 3D-N configurations had significantly higher overall immunomodulatory desirability scores relative to 2D-H and 2D-N configurations (**Figure 7A**), further corroborating that 3D-N MSC(AT) displayed augmented immunomodulatory properties. No significant differences in overall immunomodulatory desirability scores could be detected between donors.

Similar desirability analyses were performed using the angiogenic markers that correlated with angiogenic HUVEC PC1 scores (Licensed panel: *SMAD7*, *HGF*, *ANG*, *IDO1*, *TSG101*, *CXCL8*, VEGF protein; Unlicensed panel: *SOX9*, *TNFAIP6*, VEGF protein). The analysis revealed that donor differences dominated over the desirability rankings with Donors 1, 2, and 3 displaying significantly higher scores relative to Donor 4 (**Figure 7B**), further suggesting that the effect of donor predominated over variations in CPP conditions when considering MSC(AT) angiogenic functionality. Donor 5 was excluded from the desirability analysis due to an incomplete dataset. No significant differences were observed for overall angiogenic desirability scores across variations in CPP conditions.

Altogether, using the matrix of putative CQAs that included MSC(AT) genes and soluble factors, morphological features, and immunomodulatory/angiogenic functional readouts, desirability analysis allowed us to empirically rank MSC(AT) immunomodulatory and angiogenic fitness across varying CPP conditions that enhance immunomodulation and angiogenic potency, and across MSC(AT) donors. The results showed that MSC(AT) cultured under 3D-N conditions displayed the highest overall immunomodulatory ranking, while specific adipose tissue donors had highest angiogenic desirability rankings.

## Discussion

In the present study, we selected a matrix of putative quantitative CQAs with a range of minimum and maximum values that define MSC(AT) immunomodulatory and angiogenic basal fitness *in vitro* and are sensitive enough to detect variations in CPPs and donor heterogeneity. We generated putative CQAs based on statistically significant or near-significant correlations between MSC(AT) genes, soluble factors, and morphometric features and functional anchor *in vitro* readouts for immunomodulation (polarization of MΦs to pro-resolving subtypes) and angiogenic potency (network of tube formation with HUVECs). Importantly, the putative CQAs can empirically rank the relative immunomodulatory or angiogenic fitness of MSC(AT) across varying CPP conditions and donors to identify: i) optimal cell culture conditions, or ii) optimal MSC(AT) donors with desired functionality. Our approach is substantially more rigorous than use of surface identity markers which are frequently used as potential final product release criteria for MSCs therapeutics, despite clarifications made by the ISCT and the FDA (55, 56) and given that these surface markers are not sensitive to variations in donor or culture conditions (57). Our approach is also aligned with recommendations from the ISCT to characterize MSC fitness using a matrix of assay readouts (58, 59).

The CPP conditions we investigated had a pronounced effect on immunomodulatory fitness as measured by *in vitro* MSC(AT)-mediated MΦ polarization, with transient 3D-N conditions best augmenting MSC(AT) immunomodulatory basal functionality. We showed strong upregulated expression of immunomodulatory genes (*TNFAIP6*, *ICAM1*, and *PRG4*) and soluble factors (HGF, TGF-β, IL-1RA) by MSC(AT) cultured under 3D-N conditions. Our data also corroborates previous work that showed 3D MSC(M) spheroids promoted an inflammation-resolving macrophage phenotype *in vitro* and suppressed inflammation in a mouse model of peritonitis (31, 60). In contrast to our work using xeno-free 3D culture conditions, others have shown that 3D MSC(M) spheroids lose their ability to suppress pro-inflammatory macrophage activities *in vitro* when cultured using xeno-free medium (36). These differences may be attributed to different methods used for generating 3D cell aggregates. We also investigated the combination of 3D and hypoxic culture and found no additive effects using a targeted panel of both immunomodulatory and angiogenic genes. Based on this data, we evaluated 3D-N and 2D-H conditions separately recognizing that these are currently being explored as CPPs that enhance MSC(AT) fitness. Nevertheless, our methodological approach provides a platform for other investigators looking to evaluate and optimize different CPP conditions individually or in combinations using the provided range of quantitative CQAs.

MSC(AT) cultured under 2D-H conditions showed similar gene/soluble factor expression profiles relative to 2D-N conditions, with upregulation of only a few select immunomodulatory markers (*PTGS2* and PD-L2), and concordantly exhibited intermediate immunomodulatory functions. There is limited data on the immunomodulatory functions of MSCs cultured under hypoxic conditions, but previous work has shown that hypoxic culture can augment T cell inhibition mediated by rat MSC(M) (30) and human MSC(AT) (29). While varying CPP conditions exerted a greater effect on *in vitro* immunomodulation, increased expression of some pro-inflammatory MΦ markers was observed in co-cultures with MSC(AT) and this was partly donor-driven. Previous work has shown that MSCs can induce a mixed MΦ phenotype which may be important for augmenting MΦ microbicidal functions (61), and our work suggests an effect of donor heterogeneity on inducing these mixed MΦ phenotypes.

In contrast to results measuring immunomodulatory fitness, effects of donor heterogeneity predominated over effects of variations in CPPs in modulating angiogenic basal functionality of MSC(AT). This result is surprising given previous work that has shown augmented angiogenic functions of MSC(AT) cultured in 3D aggregates (32, 34), and extensive literature showing augmented angiogenic functions of MSC(AT) cultured under hypoxic conditions (27, 28, 35). Culture under 2D-H conditions augmented pro-angiogenic functions of MSC(AT) only for select donors. This discrepancy with prior literature could be related to the transient (16-20 h) incubations used for 3D/hypoxic priming. Furthermore, differences in the 3D culture method used in this study (e.g., culture using xeno-free medium), and a relatively high level of hypoxic oxygen tension (38 mmHg or approximately 5% O_2_) could account for differences relative to previous work. Nonetheless, our data demonstrated a significant effect of donor heterogeneity and suggests that this predominates over variations in CPPs in dictating MSC(AT) basal angiogenic functionality. In future work, addition of specific pro-angiogenic factors (such as FGF-2 (17)) could be explored in conjunction with the CPP conditions in a strategy analogous to use of the pro-inflammatory licensing factors to induce additional expression of angiogenic genes/proteins.

The selection of *in vitro* functional MSC(AT) readouts were carefully chosen to anchor the matrix of multivariate read-outs and refine putative CQAs according to two therapeutically relevant properties of MSCs: immunomodulation and angiogenesis. In terms of immunomodulatory functions, MSC(AT)-mediated MΦ polarization toward pro-resolving subtypes was evaluated, recognizing that MΦs represent primary effector cells for mediating MSC therapeutic functions in multiple diseases, including GVHD (9), colitis (62), and osteoarthritis (8). While effects of MSCs on T cells are more frequently employed as an *in vitro* readout to evaluate MSC immunomodulatory potency, this is not a gold standard as previous work has also demonstrated lack of correlation to clinical efficacy of MSC(M) in GVHD patients (10). Angiogenic functionality of MSC(AT), was measured using the widely-accepted HUVEC tube formation read-out, which recapitulates several aspects of *in vivo* angiogenesis including endothelial cell adhesion, migration, alignment, and formation of tubules (54). This assay has also been used to evaluate angiogenic functionality for clinical-grade MSCs (19, 38).

The functional *in vitro* read-outs were used as anchors in the putative CQA matrix allowing refinement of a putative set of 60 CQAs (48 genes of interest, 10 soluble factors, and 2 morphometric features) down to 20 CQAs that correlated to *in vitro* MΦ polarization, and 10 putative MSC(AT) CQAs that correlated to *in vitro* HUVEC tube formation. Interestingly, angiogenic genes measured under both licensed and unlicensed conditions negatively correlated with MΦ polarization toward inflammation-resolving subtypes. Conversely, increased expression of select immunomodulatory genes were also negatively correlated to greater *in vitro* HUVEC tube formation. This data suggests an inverse interplay between MSC(AT) immunomodulatory and angiogenic fitness and corroborates previous work by Boregowda *et al.* (17, 63). Our analysis supported the utility of previously reported MSC characteristics that correlate to immunomodulatory fitness – including T*NFAIP6* expression (encoding for TSG6) (8, 64, 65), *TGFB1* expression (8), and cell diameter (15)); and to angiogenic fitness, including increased VEGF expression (38). Furthermore, our results suggest that different CPP conditions or MSC(AT) donors should be selected/optimized for clinical applications depending on the target disease indication and desired therapeutic mechanism.

We applied desirability analysis to empirically rank the immunomodulatory or angiogenic basal fitness of each MSC(AT) donor or CPP condition. The results mirrored the functional assay outcomes, demonstrating a greater effect of CPPs on MSC(AT) immunomodulatory functions, and a greater effect of donor heterogeneity on angiogenic functions. Notably, we selected MSC(AT) characteristics (genes, soluble factors, and morphological features) for inclusion in the desirability analysis based on the strength of their correlations to the functional anchor assays, and we used the R^2^ goodness-of-fit values as a relative “importance ranking” for each of the MSC(AT) characteristics. Thus, MSC(AT) characteristics with significant correlations to functional assay outcomes (lower p value and higher R^2^ value) contributed to the overall desirability score to a larger extent compared to characteristics with near-significant correlations (higher p value and lower R^2^ value). Based on these analytical methods, it is expected that the overall desirability rankings would closely match the results from the original functional assay outcomes as they were used to determine the relative strength of contributions to the overall desirability score calculations. Nonetheless, our empirical approach has utility in future studies where similar desirability analysis can be used as an unbiased tool to rank the immunomodulatory or angiogenic fitness of a given population of MSCs based on combinatorial analysis of a matrix of curated genes, soluble factors, and morphological features only. The same relative importance rankings for the set of putative CQAs could be applied, circumventing the need to conduct lengthy, non-high-throughput functional analyses. To our knowledge, this is the first reported application of desirability analysis for understanding MSC potency attributes.

In the present study, we used *in vitro* functional assays as surrogate read-outs for MSC(AT) immunomodulatory and angiogenic fitness. These analyses generated relatively broad panels of putative CQAs selected based on correlation analyses to *in vitro* functional readouts with a p value threshold set to p<0.1. We have, necessarily, termed the CQAs we evaluated here as “putative”; ultimately these putative CQAs can be narrowed down further and validated in clinical studies where specific MSC(AT) CQAs may be more or less relevant depending on the target disease and/or disease stage. To accelerate clinical translation of MSC products, there is a need for simple, robust, and reproducible potency readouts, which our panel of quantitative and correlative CQAs supply. Importantly, we used two *in vitro* functional readouts as surrogate anchors for clinical efficacy. Future studies may substitute disease-specific biomarkers as recommended by Krampera and Le Blanc (3) *in lieu* of the *in vitro* functional readouts; correlation of a refined list of MSC CQAs with changes in disease attributes would be the ultimate validation. In this vein, creation of registry databases that would allow logging of large datasets, including our proposed CQA matrix used to characterize MSC(AT), may be practical to allow different sponsors to query candidate CQAs in their respective clinical MSC products and examine correlations with disease-specific biomarker changes and thus therapeutic efficacy.

Variable clinical responses to MSC treatments can arise from several factors, including through heterogeneity in donor or CPP factors explored here, but also through *in vivo* interactions with host tissues (3, 5). While we are unable to capture host responses using the *in vitro* assays applied in this work, we argue that defining basal thresholds of MSC immunomodulatory and/or angiogenic fitness with defined ranges of quantitative CQAs enables a greater likelihood of eliciting stronger therapeutic effects, as hypothesized by the ISCT MSC Committee (66). MSC basal fitness levels are especially relevant to therapeutic potency for extravascular delivery where *in vivo* persistence allows for release of paracrine factors (66), compared to vascular delivery where efficacy may rely more on proclivity of MSCs to be rapidly cleared by host immune cells as evidenced in GVHD by Galleu *et al.* (9). Another limitation of our study design was the sourcing of MSC(AT) from patients with knee or hand osteoarthritis which was based on tissue availability in our research center. It should be noted that these MSCs satisfied minimal surface marker expression criteria for MSC(AT) (42), and the adipose tissue depots were located external to the affected joints from patients with mild to moderate osteoarthritis (KL grade 0-2). However, both local joint factors and systemic factors can contribute to the pathology of osteoarthritis (67), and thus we cannot discount potential effects on the isolated MSC(AT). Ultimately, our findings will need to be verified with additional MSC(AT) donors in the context of a registry of clinical studies to evaluate whether MSC(AT) with putative CQAs within the proposed ranges perform more effectively.

Taken together, our study provides a systematic, empirical approach to evaluate the effects of variations in CPPs, specifically those that can non-genetically enhance MSC immunomodulatory and/or angiogenic properties, and donor heterogeneity on MSC(AT) critical attributes. We established a matrix of putative, quantitative CQAs with a range of minimum and maximum values based on correlations of multivariate readouts of MSC(AT) cell morphology, gene expression, and soluble factor expression with functional readouts that served as an anchor in the analysis. We argue the relevance of these functional assays in determining CQAs for MSC(AT). Importantly, the empirical approach can be adapted and applied to future clinical studies where changes to disease-specific biomarkers may be substituted *in lieu* of *in vitro* functional readouts to serve as the anchor. Our putative CQA matrix empirically ranked the effects of CPPs or donor heterogeneity on desired immunomodulatory or angiogenic MSC(AT) basal fitness, and showed differential sensitivity to these variables, suggesting that a one-size-fits-all approach is not suitable for manufacturing MSC(AT). Ultimately, our analysis identified putative CQAs that may be used to prospectively screen potent MSC(AT) donors and select specific CPP conditions to enhance for desired MSC basal fitness ranges.

## Data availability statement

The data presented in the study are deposited in the Gene Expression Omnibus repository, accession number GSE212368.

## Ethics statement

The studies involving human participants were reviewed and approved by University Health Network. The patients/participants provided their written informed consent to participate in this study.

## Author contributions

All authors contributed to conceptualization and design of the study. KR performed the experiments and analysed the data. The manuscript was drafted by KR and SV. All authors contributed to the article and approved the submitted version.

## Funding

This research was funded by the Canadian Institutes of Health Research (CIHR) (PJT-166089), the Natural Sciences and Engineering Research Council of Canada (NSERC) (RGPIN-2018-05737), and an Ontario Institute for Regenerative Medicine (OIRM) New Ideas Grant awarded to SV. The work is in part supported by the Schroeder Arthritis Institute *via* the Toronto General and Western Hospital Foundation (University Health Network). Salary support for KR was provided by a Natural Sciences and Engineering Research Council (NSERC) Canada Graduate Scholarship, an Ontario Graduate Scholarship, and by the Arthritis Society (TGP-18-0206).

## Acknowledgments

The authors would like to thank Dr. Heather Baltzer, Daniel Antflek, Kim Perry, Mary Nasim, and Luis Montoya for their assistance with adipose tissue acquisition. We would also like to acknowledge the work of Dr. Pamela Plant and Dr. Mozhgan Rasti for their technical assistance in running the NanoString gene expression and western blot experiments, respectively. The human umbilical vein endothelial cells used in this study were kindly gifted by Dr. Edmond Young. Flow cytometry for LEGENDplex experiments was performed in the Toronto Western KDT-UHN Flow Cytometry Facility, with funding from the Canada Foundation for Innovation, and Toronto General and Western Hospital Foundation. Schematics for figures were created using BioRender.com.

## Conflict of interest

SV declares 60% ownership in Regulatory Cell Therapy Consultants, Inc., a private regulatory consulting company.

The remaining authors declare that the research was conducted in the absence of any commercial or financial relationships that could be construed as a potential conflict of interest.

## Publisher’s note

All claims expressed in this article are solely those of the authors and do not necessarily represent those of their affiliated organizations, or those of the publisher, the editors and the reviewers. Any product that may be evaluated in this article, or claim that may be made by its manufacturer, is not guaranteed or endorsed by the publisher.
