# Supplementary material for: Putative critical quality attribute matrix identifies mesenchymal stromal cells with potent immunomodulatory and angiogenic “fitness” ranges in response to culture process parameters
Source: Front Immunol. 2022 Nov 30;13:972095. doi: 10.3389/fimmu.2022.972095 (PMC9747767; doi:10.3389/fimmu.2022.972095)
Supplement: Supplementary file 1 [file DataSheet_1.pdf]

Supplementary Material

1 Supplementary Figures:

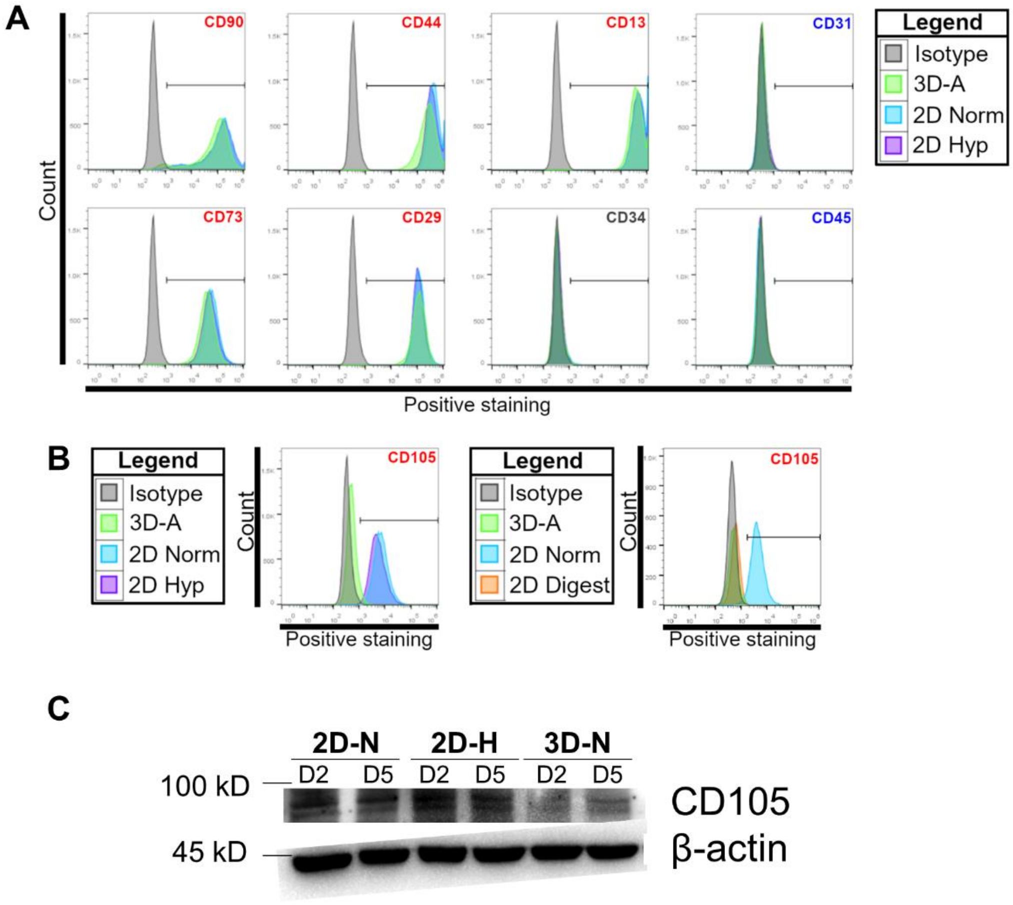

Supplementary Figure 1. MSC(AT) cultured using 3D-N and 2D-H conditions satisfied surface marker expression criteria by IFATS and ISCT [42]. A) Single cell suspensions of MSC(AT) display expression of positive MSC(AT) markers (red font) and lack expression of the variable marker CD34 as well as hematopoietic markers (blue font). B) Enzymatic digestion conditions used for dissociating 3D-N MSC(AT) cleave CD105 from both 3D-N and 2D-N MSC(AT) (2D Digest condition in right plot). Representative plots from N=3 MSC(AT) donors (two donors from subcutaneous knee fat, one abdominal lipoaspirate donor). C) Western blot demonstrating expression of CD105 by MSC(AT) cultured using 2D-N, 2D-H, and 3D-N methods. N=2 donors. D: Donor, 3D-N: 3D Normoxic culture, 2D-N: 2D Normoxic culture, 2D-H: 2D Hypoxic culture.

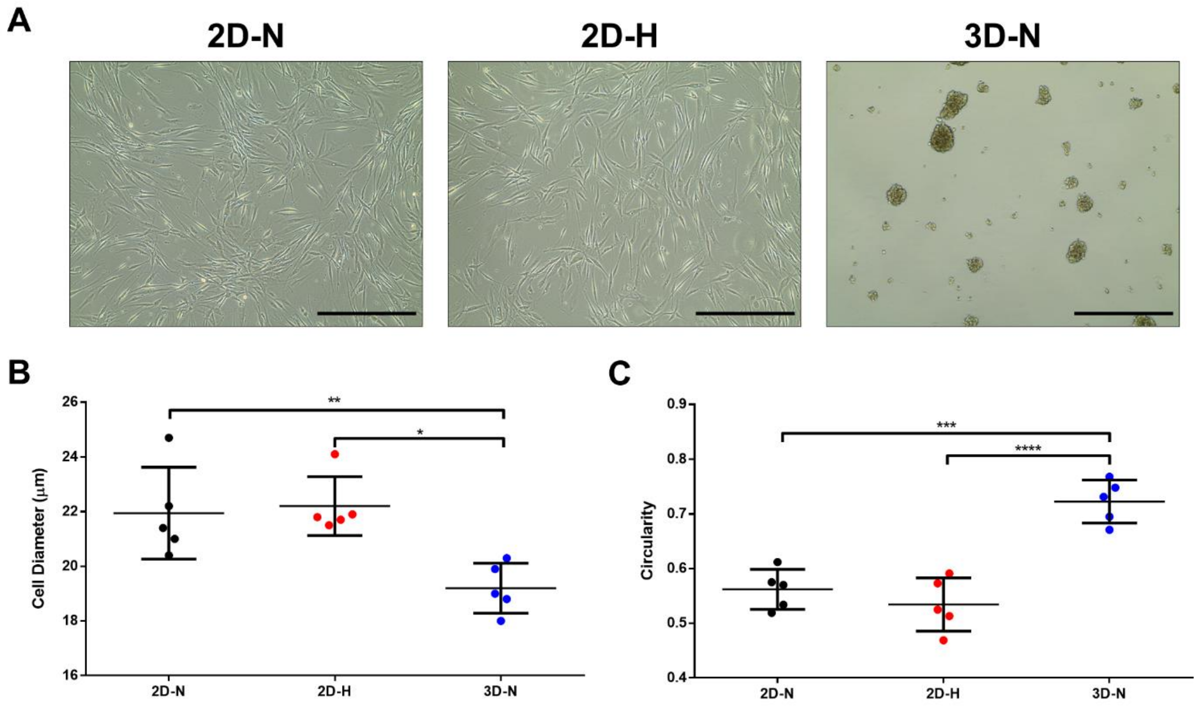

Supplementary Figure 2. Morphometric characterization of MSC(AT) cultured under various CPP conditions known to enhance immunomodulatory and/or angiogenic MSC potency. A) Representative photomicrographs of MSC(AT) cultured using each CPP. Scale bars: 500 µm. B and C) Dissociated 3D aggregates showed reduced single cell diameter (B) and greater circularity (C) relative to 2D-N and 2D-H conditions. One-way ANOVA, Tukey post-hoc test. *p<0.05, **p<0.01, ***p<0.001, ****p<0.0001. N=5 MSC(AT) donors, n=3 technical replicates. Horizontal bars: group mean, error bars: standard deviation. 3D-N: 3D Normoxic culture, 2D-N: 2D Normoxic culture, 2D-H: 2D Hypoxic culture.

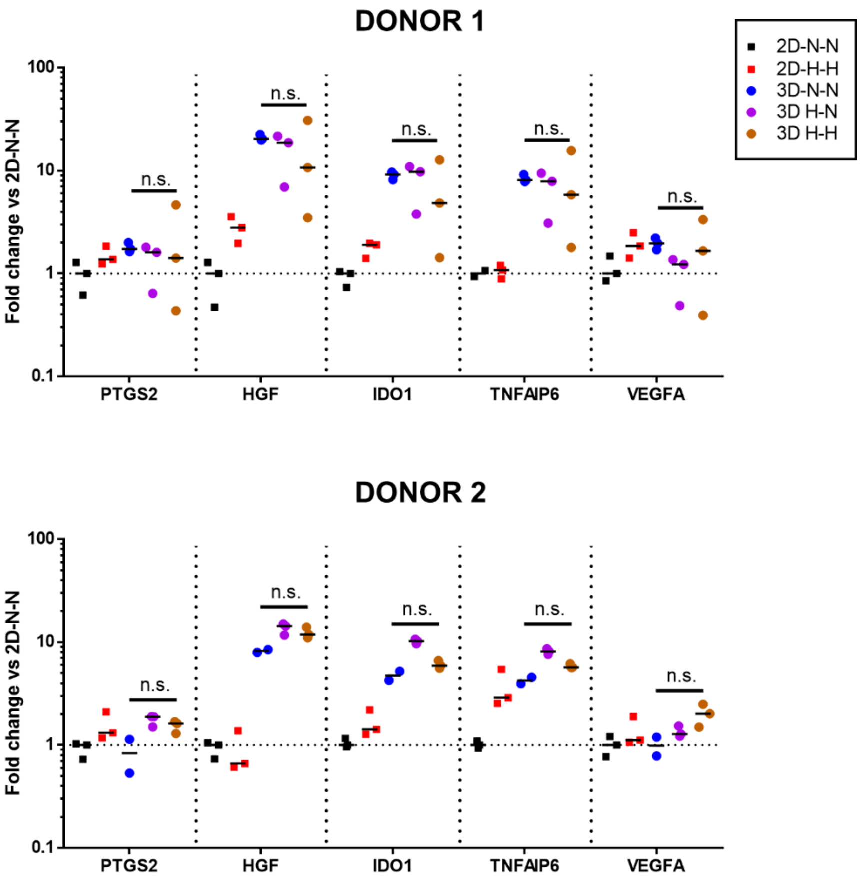

Supplementary Figure 3. Gene expression analysis of licensed MSC(AT) demonstrated no advantage of combining 3D and hypoxic culture conditions. Two MSC(AT) donors (D1 and D2) were cultured overnight (16-20 h) using 2D or 3D conditions with normoxic or hypoxic culture, followed by an additional 24 h of 2D or 3D culture in normoxic or hypoxic conditions with addition of pro-inflammatory licensing factors prior to harvesting samples for gene expression analysis by qPCR. 2D/3D indicates culture under 2D or 3D conditions. First "N" or "H" denotes culture under normoxic (N) or hypoxic (H) conditions during the initial 2D/3D culture period. Second "N" or "H" denotes culture under normoxic (N) or hypoxic (H) conditions during the licensing periods. Two-way ANOVA, Tukey post-hoc test. n.s.: statistically non-significant comparisons between 3D groups indicated. Horizontal bars: group median. N=2 MSC(AT) donors, n=3 technical replicates.

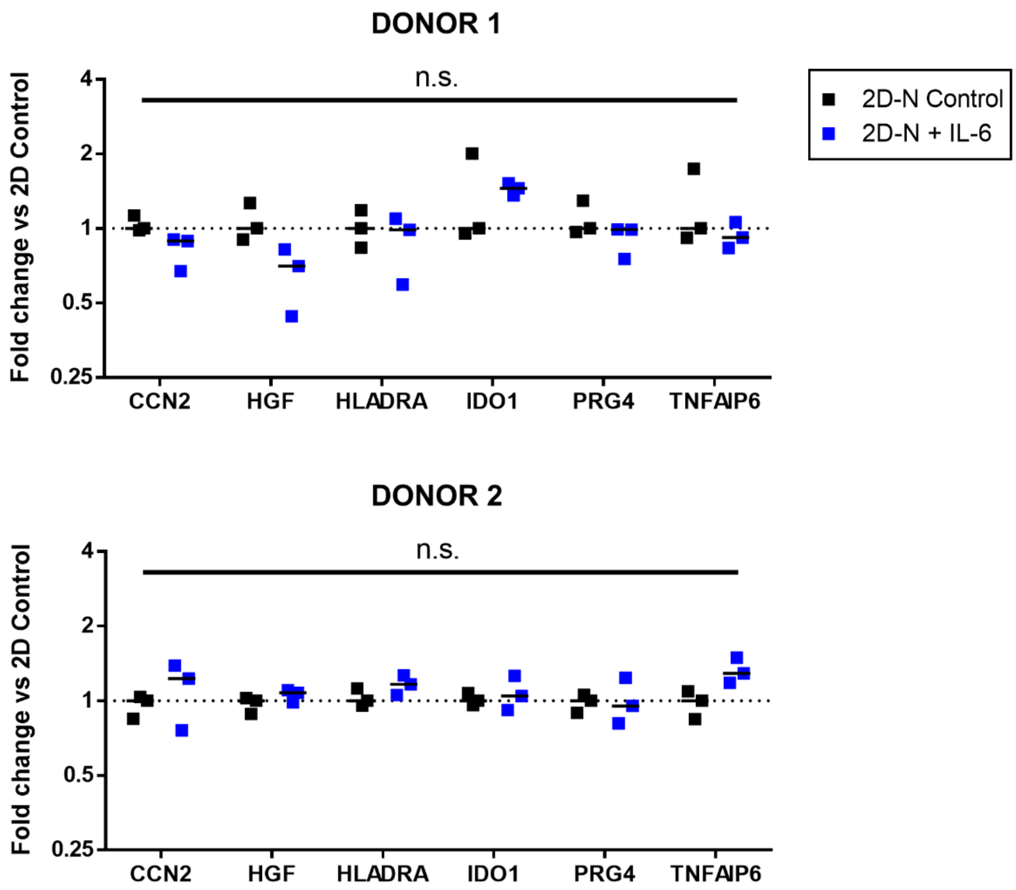

Supplementary Figure 4. Gene expression analysis of licensed MSC(AT) demonstrated no effect of IL-6 treatment on MSC(AT) cultured under 2D-N conditions. Two MSC(AT) donors cultured under 2D-N conditions with addition of IL-6 (at same concentration and duration as used for 3D-N culture) showed no significant changes in a panel of genes that were differentially expressed by MSC(AT) cultured under 3D-N conditions. Two-way ANOVA, Tukey post-hoc test. n.s.: statistically non-significant. 2D-N Control: 2D Normoxic culture without IL-6 treatment. Horizontal bars: group median. N=2 MSC(AT) donors, n=3 technical replicates.

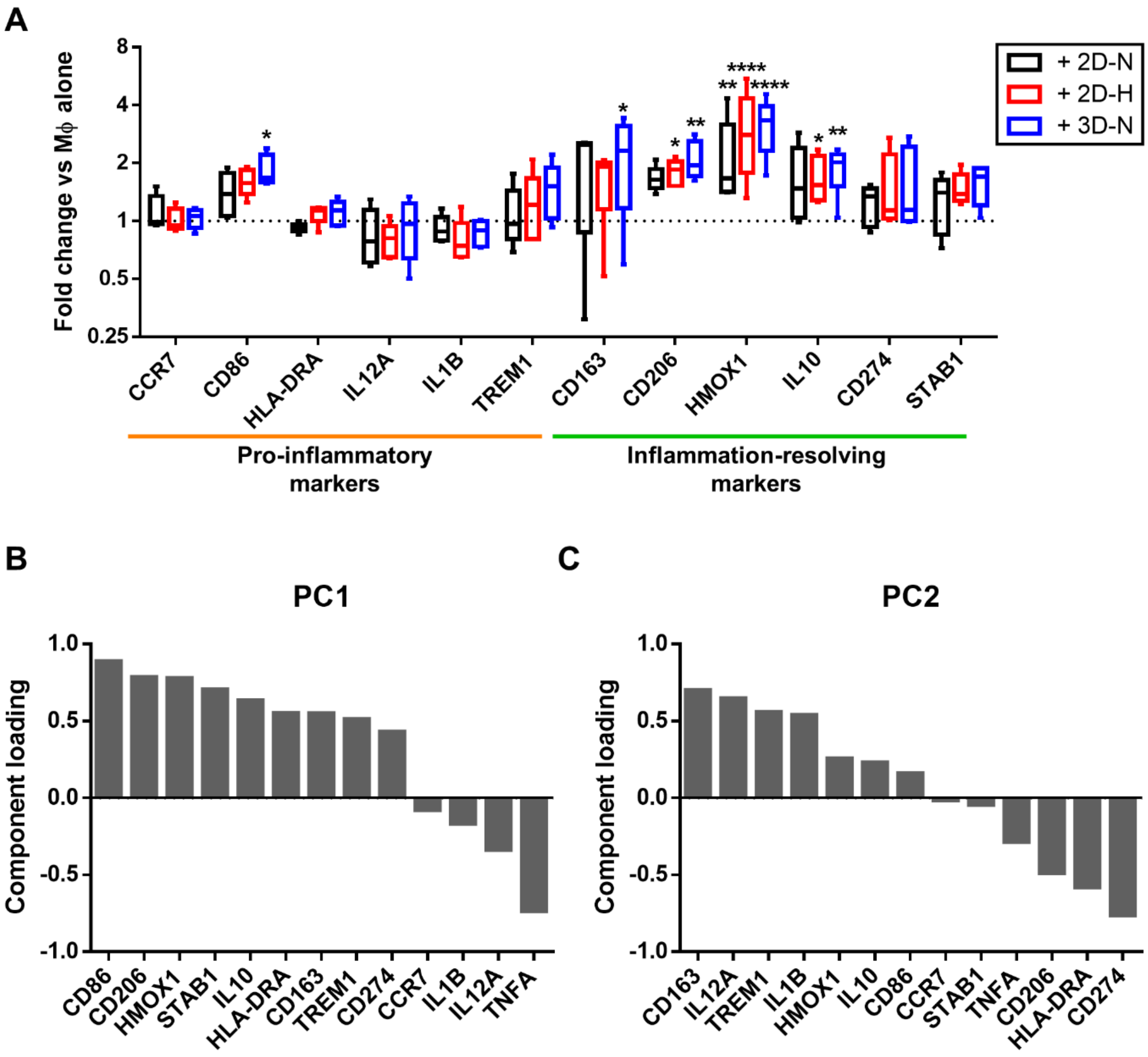

Supplementary Figure 5. MSC(AT)-mediated MΦ polarization. A) Box-and-whisker plots display changes in gene expression relative to MΦ cultured alone. Two-way ANOVA, Tukey post-hoc test. *p<0.05, **p<0.01, ****p<0.0001 vs MΦ cultured alone. 3D-N: 3D Normoxic culture, 2D-N: 2D Normoxic culture, 2D-H: 2D Hypoxic culture. N=5 MSC(AT) donors, n=3 technical replicates. B and C) Factor loading plot shows the relative contribution of each factor to principal component 1 (PC1) (B) and PC2 (C) corresponding to the PC analysis in Figure 4C. Factors are listed in order of decreasing loadings. Loadings close to ±1 have a greater relative contribution to the respective PC.

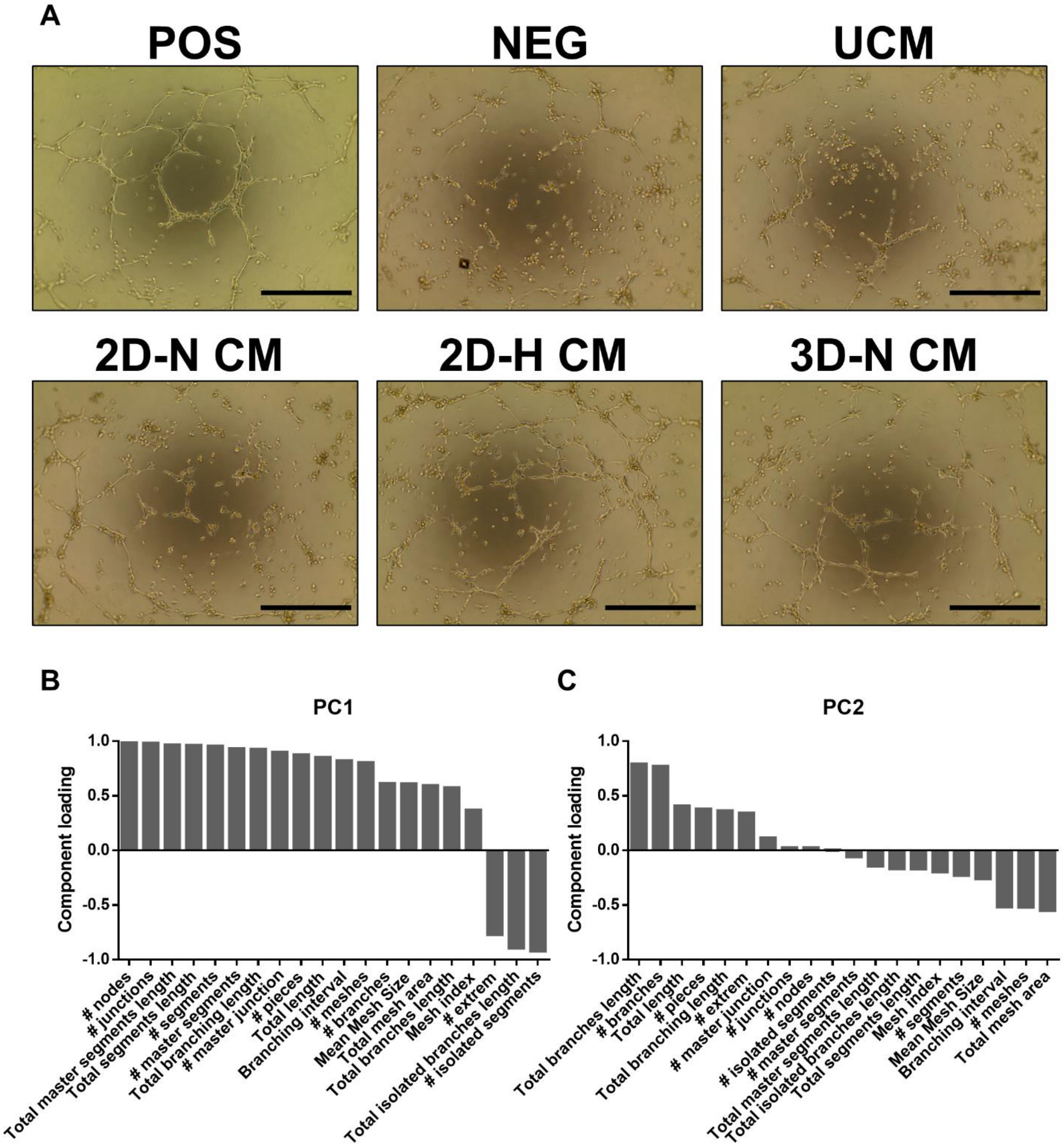

Supplementary Figure 6. HUVEC tube formation analysis. A) Phase-contrast images of HUVEC tube formation assay. Scale: 500 μm. POS: positive control, NEG: negative control, UCM: unconditioned medium, 3D-N: 3D Normoxic culture, 2D-N: 2D Normoxic culture, 2D-H: 2D Hypoxic culture, CM: conditioned medium. B and C) Factor loading plot shows the relative contribution of each factor to principal component 1 (PC1) (B) and PC2 (C) corresponding to the PC analysis in Figure 5B. Factors are listed in order of decreasing loadings. Loadings close to ±1 have a greater relative contribution to the respective PC.

A

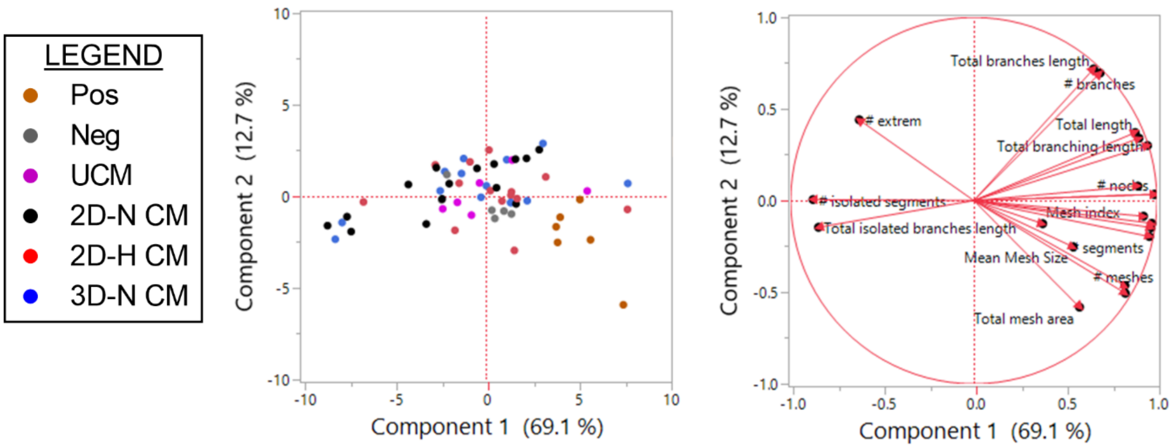

B

Donor	Media	PC1 Score	Mean PC1 Score ± SD
	Positive Control	7.504	5.086 ± 2.095
		3.918	
		3.836	
	Negative Control	0.331	-0.363 ± 1.802
		-2.408	
		0.990	
	UCM Control	-1.552	-1.562 ± 0.785
		-0.782	
		-2.351	
D2	2D-N	-3.247	-3.284 ± 0.922
		-4.224	
		-2.382	
	2D-H	1.558	1.441 ± 0.102
		1.367	
		1.401	
	3D-N	0.034	-0.359 ± 1.962
		-2.487	
		1.377	
D4	2D-N	-7.571	-7.850 ± 0.688
		-8.633	
		-7.346	
	2D-H	-6.674	-4.065 ± 3.691
		-1.455	
	3D-N	-7.837	-8.024 ± 0.265
		-8.212	

C

Donor	Media	PC1 Score	Mean PC1 Score ± SD
	Positive Control	5.132	4.967 ± 0.840
		4.056	
		5.712	
	Negative Control	-2.122	-0.0759 ± 1.829
		1.401	
		0.493	
	UCM Control	5.535	2.209 ± 3.014
		-0.343	
		1.436	
D1	2D-N	-1.995	1.047 ± 2.657
		2.915	
		2.221	
	2D-H	-0.863	-0.555 ± 1.311
		0.883	
		-1.685	
	3D-N	7.747	4.002 ± 3.390
		1.143	
		3.116	
D3	2D-N	-2.701	-0.520 ± 2.162
		1.622	
		-0.480	
	2D-H	0.169	0.228 ± 3.014
		3.270	
		-2.756	
	3D-N	2.258	0.223 ± 1.841
		-1.326	
		-0.262	
D5	2D-N	1.652	0.894 ± 0.659
		0.581	
		0.449	
	2D-H	7.728	3.225 ± 3.967
		1.703	
		0.244	
	3D-N	-1.207	-0.695 ± 1.853
		-2.239	
		1.360	

Legend on following page

Supplementary Figure 7. Principal component analysis of HUVEC tube formation image analysis read-outs showed differential effects of experimental batch, MSC(AT) donor heterogeneity, and variations in CPP conditions. A) Principal component (PC) analysis of fold-change values (relative to negative control) of twenty HUVEC tube formation image analysis read-outs, including all biological and technical replicates (left) with corresponding loading plot of eigenvectors for each measurement (right). B and C) PC1 scores corresponding to technical replicates for each culture condition and donor within experimental batch 1 (B) and batch 2 (C). N=5 MSC(AT) donors, n=2-3 technical replicates/condition. Horizontal bars: group mean, error bars: standard deviation. Pos: positive control, Neg: negative control, UCM: unconditioned medium, 3D-N: 3D Normoxic culture, 2D-N: 2D Normoxic culture, 2D-H: 2D Hypoxic culture, CM: conditioned medium. Note, this data includes biological and technical replicates while Figure 5 is based on median values of biological replicates only.

2 Supplementary Tables:

Supplementary Table 1. Forward and reverse primer sequences for qPCR.

Gene	Forward	Reverse
ACTB	AGAGGGAAATCGTGCGTGAC	AGGAGCCAGGGCAGTAATC
B2M	CTCCGTGGCCTTAGCTGTG	TTTGGAGTACGCTGGATAGCCT
CCN2	TGTGGCTTTAGGAGCAGTGG	GCTACAGGCAGGTCAGTGAG
CCR7	TTTTACCGCCCAGAGAGCG	AATGACAAGGAGAGCCACC
CD163	TGGACCTAATGAATTCCTCAGAAAA	ACACAGAAATTAGTTCAGCAGCA
CD206	CTACAAGGGATCGGGTTTATGGA	TTGGCATTGCCTAGTAGCGTA
CD274	TCAATGCCCCATACAACAA	TGCTTGTCCAGATGACTTCG
CD86	CTGCTCATCTATACACGGTTACC	GGAAACGTCGTACAGTTCTGTG
CD86	CCATCAGCTTGTCTGTTTCATTCC	GCTGTAATCCAAGGAATGTGGTC
HGF	TGGTTTTAATGAAGCTTGCCAG	GAGATGTGCCACTCGTAATAGG
HLADRA	AAGCACTGGGAGTTTGATGC	ATTGCTTTTGCGCAATCCCT
HMOX1	AAGACTGCGTTCCTGCTCAAC	AAAGCCCTACAGCAACTGTCG
IDO1	GCCCTTCAAGTGTTTCACCAA	CCAGCCAGACAAATATATGCGA
IL10	CGAGATGCCTTCAGCAGAGT	CGCCTTGATGTCTGGGTCTT
IL12A	CCTCCACTGTGCTGGTTTTAT	TCAGCAACATGCTCCAGAAG
IL1B	GTACCTGTCCTGCGTGTTGA	GGGAACTGGGCAGACTCAAA
IL1RA	AGCATGAGGCTCAATGGGTA	AAATCCAGCAAGATGCAAGC
PRG4	AGGCCCCATGTGTTCATGC	GCGCAAAGTAGTCAGTCCATCT
PTGS2	ATAAGCGAGGGCCAGCTTTC	CGCAGTTTACGCTGTCTAGC
RPL13A	TGCGACAAAACCTCCTCCTT	TGTTGATGCCTTCACAGCGTA
STAB1	GAACCATGTGCCACTGGAAGGC	AGCGGAATCTCCTGGTGCAGTT
TBP	AGCGCAAGGGTTTCTGGTTT	AATAGGCTGTGGGGTCAGTC
TNFAIP6	AGCACGGTCTGGCAAATACA	ATCCATCCAGCAGCACAGAC
TREM1	AGTTGCAGCTCGGAGTTCTGAGACA	GAACCATGTGCCACTGGAAGGC
VEGFA	CTACCTCCACCATGCCAAGT	AGCTGCGCTGATAGACATCC

Supplementary Table 2. Gene expression measured for MSC(AT) under licensed conditions. All data values represent mRNA counts. 2D-N: 2D normoxic, 2D-H: 2D hypoxic, 3D-N: 3D normoxic, D: donor.

	2D-N				2D-H					3D-N				
	**D1**	**D2**	**D3**	**D4**	**D1**	**D2**	**D3**	**D4**	**D5**	**D1**	**D2**	**D3**	**D4**	**D5**
ACTA2	66.71	73.01	159.42	126.59	54.88	59.98	201.10	104.78	182.93	64.57	61.05	73.53	129.03	130.04
ANG	1.00	29.78	31.29	49.80	27.05	27.93	46.31	45.26	58.40	1.00	32.76	33.61	61.01	60.46
ANGPT1	69.55	68.21	215.51	60.18	77.29	50.94	237.48	105.62	79.87	42.27	61.05	204.84	94.67	104.95
CCN2	370.44	634.06	366.07	414.00	175.45	315.50	416.75	274.10	470.63	63.40	95.29	96.64	214.58	295.45
CD274	55.35	60.52	71.44	179.51	81.93	117.49	160.09	202.01	196.67	136.19	92.32	102.95	155.68	242.98
CXCL8	33604.11	31658.65	31881.42	51553.32	31885.05	25841.33	44656.26	49225.02	46010.56	26878.33	31301.07	27422.28	37158.81	25008.42
EDIL3	33.35	268.03	240.90	44.62	38.64	188.15	302.97	64.54	287.71	149.10	564.32	571.45	122.02	400.40
EDN1	1.00	28.82	34.24	63.29	35.55	1.00	63.51	59.51	65.27	1.00	1.00	1.00	1.00	45.63
F3	132.70	269.95	1253.48	482.49	192.45	188.97	1602.85	781.22	1752.86	194.89	737.04	1655.53	1138.82	2779.98
HGF	139.80	373.71	774.05	1322.94	191.68	569.38	1041.89	1590.93	380.46	801.87	1926.72	2294.21	4151.35	1639.24
HLA-DRA	58.90	91.27	303.48	227.24	40.19	52.58	279.82	93.04	190.66	241.85	608.99	2322.57	1131.80	2098.96
ICAM1	1724.45	2976.22	3400.87	5953.77	2317.14	3178.82	4966.00	6604.30	7610.89	3950.62	7120.24	6305.92	8007.48	10232.43
IDO1	6089.52	6800.74	4326.67	12426.34	7418.26	6853.89	5440.30	13258.88	9205.72	12371.97	13193.74	9266.12	17824.17	14762.30
NFKBIA	1167.38	1195.10	1092.29	2003.61	1294.60	1262.00	1598.22	2450.10	2114.42	2472.52	3071.74	2177.61	3567.22	3125.62
NOS2	1.00	1.00	1.00	1.00	1.00	1.00	1.00	1.00	1.00	31.70	52.11	183.83	52.59	71.87
PDCD1LG2	267.54	244.98	190.12	329.96	258.15	227.59	288.42	231.35	322.06	79.83	47.65	59.88	63.81	95.82
PDGFA	1.00	1.00	31.88	32.17	1.00	1.00	37.71	1.00	33.49	1.00	28.29	37.82	32.26	38.79
PRG4	1.00	1.00	1.00	1.00	1.00	1.00	1.00	1.00	1.00	95.10	41.69	119.75	55.40	41.07
PTGES	965.13	890.56	1451.28	1562.63	908.93	955.53	2055.99	1638.71	1709.06	2157.87	2499.98	3088.35	3586.85	3151.86
PTGS2	709.65	865.58	1409.35	1610.36	1385.80	1561.06	3568.88	3367.11	693.93	2112.09	3566.08	2674.47	4453.59	862.40
SMAD7	268.25	275.72	217.87	504.28	265.10	194.72	267.25	398.99	320.34	363.95	454.14	298.33	502.79	436.90
TGFB1	97.93	96.07	78.53	169.13	119.03	270.31	219.62	267.39	188.94	206.63	227.81	118.70	297.33	246.40
THBS1	2619.32	7517.42	5645.69	9328.05	2541.28	6022.41	6726.29	4569.95	6013.48	860.57	1745.07	512.62	1135.31	2015.69
THBS2	943.13	333.36	1221.60	1577.16	1084.37	296.60	1349.49	1344.50	1368.96	1680.04	296.30	917.05	1403.19	1511.48
TIMP1	3559.61	2387.32	4687.42	3605.67	3938.68	2026.09	6256.61	4231.31	5595.23	7416.37	7633.94	10374.35	10542.47	12197.92
TLR2	72.38	410.22	161.78	1039.68	129.85	736.99	419.40	1317.67	1727.95	516.57	1682.53	774.19	1865.30	2882.65
TLR3	51.80	83.58	73.21	63.29	57.97	59.98	61.52	35.21	37.79	104.49	86.36	60.93	59.61	62.74

TLR4	1.00	30.74	30.11	1.00	1.00	1.00	38.37	1.00	30.06	30.52	52.11	58.83	35.06	76.43
TNFAIP6	390.31	525.50	284.00	892.34	487.70	707.41	608.59	1015.92	692.21	1266.78	2147.09	894.99	2300.07	1692.86
TSG101	297.34	338.16	311.75	437.87	272.06	285.10	421.39	424.98	420.82	407.39	400.53	352.95	488.06	473.41
TWIST1	1.00	1.00	1.00	28.02	1.00	40.26	59.54	1.00	27.48	1.00	28.29	54.62	63.81	54.76
VEGFA	300.89	215.20	210.78	373.54	319.98	234.16	389.63	517.18	453.46	231.28	226.32	130.26	258.76	198.49

Supplementary Table 3: Gene expression measured for MSC(AT) under unlicensed conditions. All data values represent mRNA counts. 2D-N: 2D normoxic, 2D-H: 2D hypoxic, 3D-N: 3D normoxic, D: donor.

	**2D-N**					**2D-H**					**3D-N**				
	**D1**	**D2**	**D3**	**D4**	**D5**	**D1**	**D2**	**D3**	**D4**	**D5**	**D1**	**D2**	**D3**	**D4**	**D5**
ACTA2	103.34	280.09	2470.80	292.80	528.41	78.54	269.85	3197.28	345.23	905.41	169.22	146.93	512.59	456.80	395.51
ANG	27.23	33.98	68.28	60.31	88.99	32.04	25.10	58.03	65.62	70.28	28.49	44.36	55.49	71.43	65.28
ANGPT1	37.01	33.98	204.19	92.05	114.98	60.95	27.79	170.28	94.15	118.52	45.76	41.58	327.12	96.34	93.44
CCN2	3211.24	6617.70	11393.39	5027.57	3347.68	970.09	3834.39	7376.29	3600.68	3836.61	426.51	586.33	289.15	452.65	334.07
CXCL8	488.08	314.89	230.04	250.74	458.33	98.01	158.68	76.10	233.96	250.81	609.55	298.01	299.38	434.37	350.71
EDIL3	32.12	256.06	763.71	66.65	378.00	31.41	242.96	725.83	85.59	475.44	37.13	212.08	887.90	58.14	355.83
EDN1	1.00	42.26	53.70	1.00	18.11	1.00	53.79	54.22	1.00	1.00	1.00	1.00	1.00	1.00	1.00
F3	300.95	237.83	1079.94	556.24	1270.24	807.36	346.95	1278.53	1015.72	1802.54	290.10	238.41	940.48	826.39	2017.25
HGF	43.29	91.15	653.66	168.22	273.26	37.07	83.38	554.60	92.25	165.37	133.82	224.55	1380.05	320.59	400.63
ICAM1	29.33	45.58	45.08	46.02	103.95	15.71	1.00	26.64	74.18	110.25	118.28	180.19	242.42	245.01	181.76
NFKBIA	191.32	223.74	325.51	280.10	384.30	559.19	259.99	315.83	548.76	569.15	305.64	313.26	479.00	498.33	559.35
PDCD1LG2	83.09	72.09	85.52	115.06	140.18	96.13	86.07	109.40	127.44	162.61	58.71	37.43	33.59	47.34	37.12
PDGFA	1.00	48.06	127.95	61.89	66.94	1.00	52.00	103.69	38.04	74.42	1.00	59.60	113.91	67.27	85.76
PRG4	1.00	1.00	60.33	1.00	1.00	1.00	1.00	42.81	1.00	1.00	102.74	29.11	497.98	60.63	78.08
PTGS2	62.14	35.63	49.06	67.45	92.14	324.83	94.13	43.76	337.62	226.01	98.43	67.92	78.86	52.32	40.96
SMAD7	111.02	140.04	322.85	207.10	244.91	94.87	90.55	253.04	204.48	224.63	249.52	246.73	448.33	427.73	450.55
SOX9	50.97	29.83	1.00	53.16	1.00	31.41	33.17	1.00	38.04	1.00	30.22	36.04	1.00	61.46	29.44
TGFB1	132.67	200.54	423.62	322.16	232.31	140.11	487.70	420.47	352.84	227.39	212.39	559.99	480.46	581.38	343.03
THBS1	4010.04	13388.71	10911.43	14430.46	7249.76	2613.10	9433.12	12694.95	15145.48	12391.80	1009.29	2080.55	575.38	6109.48	2771.16
THBS2	553.01	674.53	4893.20	868.08	2294.00	404.62	478.74	4279.85	780.81	2253.18	945.40	1388.88	6546.82	1918.56	3143.63
TIMP1	4588.19	3941.12	13179.36	7460.42	10995.12	4283.74	3655.99	11755.08	7918.45	11516.71	10140.39	11963.53	39473.71	23333.31	30155.04
TLR2	1.00	1.00	1.00	1.00	73.24	1.00	1.00	1.00	1.00	67.53	1.00	44.36	26.29	46.51	206.08
TLR3	1.00	1.00	45.08	1.00	25.20	1.00	1.00	34.25	1.00	26.18	1.00	26.34	167.94	1.00	1.00
TLR4	1.00	36.46	71.60	30.15	55.91	28.90	42.14	73.25	53.26	103.36	25.90	27.72	112.45	32.39	88.32
TNFAIP6	1.00	1.00	1.00	28.57	1.00	1.00	1.00	29.49	35.19	1.00	32.81	1.00	45.27	45.68	40.96
TSG101	238.80	299.98	599.97	532.43	504.79	209.22	284.20	560.31	454.60	463.04	255.56	339.60	579.77	533.21	463.35

TWIST1	1.00	1.00	55.69	38.88	40.95	33.30	104.89	91.32	55.16	38.59	1.00	31.88	58.41	44.85	48.64
VASH1	1.00	1.00	29.83	26.19	1.00	1.00	1.00	30.44	31.38	37.21	1.00	1.00	75.94	61.46	47.36
VEGFA	500.64	367.93	894.31	710.97	678.83	461.80	214.27	761.98	565.88	712.47	683.80	368.71	756.47	498.33	481.27

Supplementary Table 4: Differential gene expression analysis relative to 2D-N measured under licensed conditions. Multivariate linear regression, Benjamini-Yekutieli (BY) False Discovery Rate-corrected p values. Bolded font indicates p<0.05 vs 2D-N. 3D-N: 3D Normoxic culture, 2D-N: 2D Normoxic culture, 2D-H: 2D Hypoxic culture. N=5 MSC(AT) donors, n=1 technical replicate due to high sensitivity of Nanostring measurements.

	**3D-N vs 2D-N**			**2D-H vs 2D-N**		
	Log_2 Fold Change	Standard Error (Log_2)	BY p value	Log_2 Fold Change	Standard Error (Log_2)	BY p value
ACTA2	0.0416	0.187	1	-0.16	0.189	1
ANG	**0.595**	**0.178**	**0.0273**	0.0481	0.178	1
ANGPT1	0.422	0.201	0.28	0.0153	0.201	1
CCN2	**-1.61**	**0.202**	**2.09E-05**	**-0.851**	**0.199**	**0.0291**
CD274	**0.965**	**0.25**	**0.0104**	0.447	0.252	1
CXCL8	0.141	0.216	1	-0.0014	0.216	1
EDIL3	**1.9**	**0.237**	**2.08E-05**	0.165	0.244	1
EDN1	**-1.05**	**0.259**	**0.00751**	0.0257	0.23	1
F3	**1.37**	**0.24**	**0.000407**	0.255	0.242	1
HGF	**2.47**	**0.214**	**6.09E-07**	0.208	0.216	1
HLA-DRA	**2.99**	**0.192**	**4.36E-08**	**-0.879**	**0.207**	**0.0291**
ICAM1	**1.37**	**0.228**	**0.000249**	0.209	0.228	1
IDO1	**1.34**	**0.211**	**0.000147**	0.0651	0.211	1
NOS2	**6.07**	**0.454**	**1.68E-07**	1.52	0.454	0.14
NFKBIA	**1.51**	**0.199**	**3.08E-05**	0.203	0.199	1
PDGFA	1.34	0.625	0.27	-0.161	0.625	1
PDCD1LG2	**-1.65**	**0.216**	**3.07E-05**	-0.213	0.208	1
PRG4	**3.56**	**0.316**	**6.09E-07**	-0.479	0.419	1
PTGES	**1.68**	**0.173**	**2.82E-06**	0.0833	0.174	1
PTGS2	**1.93**	**0.215**	**6.33E-06**	**0.977**	**0.215**	**0.0291**
SMAD7	**0.819**	**0.232**	**0.0191**	-0.256	0.234	1
TGFB1	**1.42**	**0.189**	**3.12E-05**	**0.823**	**0.191**	**0.0291**
THBS1	**-2.18**	**0.301**	**4.10E-05**	-0.582	0.301	1
THBS2	0.421	0.212	0.336	-0.193	0.213	1
TIMP1	**1.82**	**0.178**	**1.71E-06**	0.0917	0.178	1
TLR2	**2.32**	**0.311**	**3.23E-05**	0.663	0.312	0.839
TLR3	**0.728**	**0.162**	**0.00348**	-0.502	0.181	0.309
TLR4	**1.59**	**0.249**	**0.000142**	-0.0289	0.249	1
TSG101	**0.697**	**0.164**	**0.00533**	-0.0988	0.166	1
TNFAIP6	**2.14**	**0.189**	**6.09E-07**	0.365	0.191	1
TWIST1	**1.98**	**0.28**	**4.86E-05**	0.849	0.299	0.309
VEGFA	0.0164	0.22	1	0.348	0.219	1

Supplementary Table 5: Differential gene expression analysis relative to 2D-N measured under unlicensed conditions. Multivariate linear regression, Benjamini-Yekutieli (BY) False Discovery Rate-corrected p values. Bolded font indicates p<0.05 vs 2D-N. 3D-N: 3D Normoxic culture, 2D-N: 2D Normoxic culture, 2D-H: 2D Hypoxic culture. N=5 MSC(AT) donors, n=1 technical replicate due to high sensitivity of Nanostring measurements.

	**3D-N vs 2D-N**			**2D-H vs 2D-N**		
	Log_2 Fold Change	Standard Error (Log_2)	BY p value	Log_2 Fold Change	Standard Error (Log_2)	BY p value
ACTA2	0.174	0.334	1	0.726	0.333	0.625
ANG	**0.626**	**0.163**	**0.0115**	0.23	0.17	1
ANGPT1	**0.928**	**0.227**	**0.00794**	0.463	0.231	0.704
CCN2	**-3.07**	**0.296**	**1.76E-06**	-0.181	0.295	1
CXCL8	0.918	0.453	0.266	-0.859	0.455	0.736
EDIL3	**0.701**	**0.186**	**0.0124**	0.551	0.187	0.157
F3	**0.829**	**0.205**	**0.00798**	**1.1**	**0.205**	**0.00596**
HGF	**1.76**	**0.173**	**1.76E-06**	-0.0518	0.181	1
ICAM1	**2.79**	**0.459**	**0.000344**	0.0797	0.47	1
NFKBIA	**1.29**	**0.22**	**0.000448**	**1.07**	**0.22**	**0.00876**
PDGFA	0.544	0.259	0.242	-0.00801	0.259	1
PDCD1LG2	-0.621	0.325	0.318	0.67	0.317	0.635
PRG4	**3.88**	**0.527**	**6.00E-05**	-0.854	0.527	0.952
PTGES	**3.67**	**0.64**	**0.000512**	0.0319	0.724	1
PTGS2	1	0.474	0.242	**1.99**	**0.471**	**0.017**
SMAD7	**1.53**	**0.147**	**1.76E-06**	0.122	0.153	1
SOX9	0.536	0.23	0.169	-0.143	0.244	1
TGFB1	**1.38**	**0.133**	**1.76E-06**	**0.642**	**0.135**	**0.00876**
THBS1	**-1.7**	**0.301**	**0.00056**	0.398	0.301	1
THBS2	**1.4**	**0.187**	**5.94E-05**	0.154	0.188	1
TIMP1	**2.11**	**0.167**	**5.63E-07**	0.308	0.167	0.736
TLR2	**2.64**	**0.518**	**0.00133**	0.561	0.546	1
TLR3	**1.84**	**0.513**	**0.0172**	0.027	0.54	1
TLR4	**1.14**	**0.23**	**0.00158**	**1.08**	**0.231**	**0.00876**
TSG101	**0.655**	**0.129**	**0.00133**	0.222	0.13	0.869
TNFAIP6	**1.94**	**0.306**	**0.000245**	0.612	0.331	0.736
TWIST1	**0.865**	**0.249**	**0.0205**	**1.43**	**0.243**	**0.0046**
VASH1	**1.85**	**0.265**	**9.63E-05**	0.868	0.265	0.0937
VEGFA	0.493	0.212	0.169	0.148	0.212	1

Supplementary Table 6: Soluble factor levels measured for MSC(AT) under licensed conditions. All data values are in units of pg/mL. Values represent the mean of two technical replicate wells. 2D-N: 2D normoxic, 2D-H: 2D hypoxic, 3D-N: 3D normoxic, D: donor.

Culture Condition	Donor ID	TGF-β	Ang1	VEGF	HGF	PD-L2	IL-1RA	PDGF-AA
2D-N	D1	111.42	12.38	617.80	9.83	648.56	39.40	0.45
	D2	107.33	16.98	368.48	37.00	529.37	39.40	3.07
	D3	116.26	60.40	609.28	286.55	524.20	39.40	24.42
	D4	593.73	9.10	74.35	28.65	591.77	39.40	1.64
	D5	1013.52	6.75	15.20	4.40	452.24	23.10	1.01
2D-H	D1	78.23	18.03	726.55	7.30	708.86	61.78	1.56
	D2	103.06	17.70	433.30	6.63	550.62	39.40	10.13
	D3	69.23	80.95	942.15	77.58	520.01	39.40	58.86
	D4	1738.44	22.25	64.05	179.03	807.59	83.08	3.79
	D5	2135.49	58.23	491.63	339.63	1002.03	62.10	14.66
3D-N	D1	1128.79	12.38	479.98	511.93	472.30	114.70	3.80
	D2	1856.83	14.03	325.48	3551.93	434.52	373.38	28.55
	D3	1517.53	70.70	576.18	5736.73	414.27	138.85	116.68
	D4	2750.45	40.73	104.15	8082.70	528.44	541.48	21.73
	D5	2417.04	46.03	226.15	3303.23	532.72	693.55	41.07

Supplementary Table 7: Soluble factor levels measured for MSC(AT) under unlicensed conditions. All data values are in units of pg/mL. Values represent the mean of two technical replicate wells. 2D-N: 2D normoxic, 2D-H: 2D hypoxic, 3D-N: 3D normoxic, D: donor.

Culture Condition	Donor ID	TGF-β	Ang1	VEGF	HGF	PD-L2	PDGF-AA
2D-N	D1	138.48	19.18	1035.88	8.05	955.69	1.17
	D2	85.54	14.35	350.88	20.30	427.44	5.30
	D3	96.14	70.15	919.28	185.13	507.20	48.11
	D4	289.42	18.78	250.58	7.00	532.60	2.48
	D5	448.12	42.20	617.85	15.85	519.10	11.10
2D-H	D1	675.93	20.75	782.55	20.75	828.29	0.75
	D2	752.90	29.13	420.65	135.30	534.55	6.25
	D3	890.42	123.60	1463.65	965.05	765.77	50.08
	D4	893.08	65.25	461.83	7.88	698.95	4.90
	D5	1196.66	65.90	901.58	36.08	752.62	20.96
3D-N	D1	866.31	17.75	1252.80	207.33	501.52	4.18
	D2	1098.27	13.80	555.98	449.48	424.14	44.59
	D3	1070.39	88.05	1108.13	2682.58	420.43	177.28
	D4	1292.81	34.68	817.40	509.60	420.42	25.04
